# Discovery of potential ALK inhibitors from *Glycosmis* phytochemicals via integrated machine learning and umbrella sampling approaches

**DOI:** 10.1098/rsos.261178

**Published:** 2026-11-04

**Authors:** Xuan Ha Nguyen, Xuan Duc Dau, Thi Minh Hue Nguyen, Ngoc Dung Tran, Van Trang Nguyen, Le Binh Doan, Thi My Chau Le, Thuy Phan Thi

**Affiliations:** ^1^ Institute of Chemistry, Vietnam Academy of Science and Technology (VAST), 18 Hoang Quoc Viet, Nghia Do, Hanoi, Vietnam; ^2^ Department of Chemistry, Vinh University, 182 Le Duan, Truong Vinh, Nghean 43000, Vietnam; ^3^ Faculty of Chemistry and Center for Computational Science, Hanoi National University of Education, 136 Xuan Thuy, Hanoi 10000, Vietnam; ^4^ Institute of Materials Science, VAST, 18 Hoang Quoc Viet, Hanoi 10000, Vietnam; ^5^ Institute of Biology, Chemistry and Environment, Vinh University, 182 Le Duan, Truong Vinh, Nghean 43000, Vietnam

**Keywords:** Anaplastic lymphoma kinase, machine learning, *Glycosmis* phytochemicals, virtual screening, umbrella sampling

## Abstract

Anaplastic lymphoma kinase (ALK) is a key therapeutic target for various cancers because of its central role in tumor growth and survival signaling. This study employed an integrated computational workflow combining machine learning (ML), molecular docking, molecular dynamics (MD) simulations, umbrella sampling (US), and toxicity prediction to identify potential ALK inhibitors from 307 phytochemicals of the *Glycosmis* genus. A previously validated CatBoost regression model predicted binding free energies, leading to the selection of 26 compounds with Δ*G*_ML_ < −11 kcal mol^−1^. Molecular docking identified six top candidates with favorable binding affinities ranging from −5.856 to −9.667 kcal mol⁻¹, which subsequently underwent 200 ns MD simulations. Structural analyses confirmed the stability of all ALK–ligand complexes, while interaction studies revealed extensive hydrophobic contacts and conserved hydrogen bonds involving key ALK residues. US calculations estimated binding free energies from −8.354 to −13.272 kcal mol^−1^, with compound 54 exhibiting the strongest affinity (Δ*G*_US_ = −13.272 kcal mol^−1^). Several alkaloids, including acrifoline, heptazoline, and atalaphyllidine, also demonstrated promisingbinding properties and reported anticancer activities. Toxicity prediction indicated acceptable safety profiles, highlighting *Glycosmis*-derived phytochemicals as promising potential ALK inhibitor candidates and supporting the utility of ML-guided virtual screening combined with advanced molecular simulations for natural product-based anticancer drug discovery. Nevertheless, biochemical and cellular experiments are required to confirm their ALK inhibitory activity, selectivity, and therapeutic potential.

## 1. Introduction

*Glycosmis* is an important medicinal plant belonging to the family Rutaceae [1]. According to the World Flora Online database, the genus comprises approximately 51 accepted species and 22 varieties distributed throughout tropical and subtropical regions [2]. Traditional and folk medicine systems have used *Glycosmis* species to treat numerous human ailments. Different plant parts, including the leaves, roots, stems, bark, and fruits, contribute to the treatment of fever, cough, inflammation, rheumatism, anemia, jaundice, liver disorders, intestinal worm infections, wounds, eczema, skin diseases, and cancer-related conditions [3–8]. Several species also demonstrate anti-venom, vermifuge, wound-healing, and hepatoprotective applications, highlighting the ethnopharmacological importance of this genus [7]. Phytochemical investigations have revealed remarkable chemical diversity within *Glycosmis* species. To date, researchers have isolated and characterized more than 300 phytochemicals from different members of this genus. These compounds include alkaloids, flavonoids, terpenoids, phenolics, sulfur-containing amides, quinones, and several miscellaneous metabolites [9–12]. Among these classes, alkaloids constitute the most abundant and pharmacologically significant group, including the acridone, carbazole, quinoline, quinolone, quinazoline, indole, and furoquinoline subclasses. Carbazole and acridone alkaloids have attracted substantial scientific attention because of their pronounced anticancer, antiproliferative, antimicrobial, antiviral, and anti-inflammatory activities.

Numerous compounds isolated from *Glycosmis* species exhibit promising anticancer potential against various human cancer cell lines [8,13–16]. Arborinine, glycoborinine, glycozolidine, glybomine B, glycopentalone, and biscarbalexine A have demonstrated significant cytotoxic and antiproliferative activities in previous experimental studies [17–19]. Dimeric carbazole alkaloids, such as glycosmisines A and B, also exhibit potent anticancer properties [20,21]. Furthermore, several *Glycosmis*-derived metabolites inhibit cancer-associated molecular targets, including protein kinase C and cyclooxygenase-2 (COX-2), suggesting considerable therapeutic potential for targeted cancer therapy [6,22]. In addition to alkaloids, flavonoids and terpenoids isolated from this genus display antioxidant, anti-inflammatory, and apoptosis-inducing activities that further strengthen the medicinal value of *Glycosmis* phytochemicals [14,23–28].

Despite the remarkable chemical diversity and broad pharmacological activities reported for *Glycosmis* species, only a limited number of compounds have been systematically evaluated against the specific molecular targets associated with cancer progression. The structural diversity of *Glycosmis* metabolites, particularly carbazole and acridone alkaloids, provides a valuable reservoir of lead-like scaffolds for anticancer drug discovery. Several carbazole-based compounds have already received approval as anticancer agents, further supporting the possibility that structurally related natural products from *Glycosmis* may serve as promising therapeutic candidates.

Anaplastic lymphoma kinase (ALK) functions as an important oncogenic target involved in cell proliferation, survival and differentiation signaling pathways [29–31]. Genetic alterations involving ALK, including gene rearrangements, amplifications, and activating mutations, contribute significantly to the development of multiple cancers, such as non–small-cell lung cancer, neuroblastoma, and anaplastic large cell lymphoma [32–35]. Although currently available ALK inhibitors provide considerable clinical benefits, acquired drug resistance and adverse side effects continue limiting their long-term therapeutic effectiveness [36,37]. Consequently, the discovery of novel ALK inhibitors with improved potency and safety profiles remains an important objective for targeted cancer therapy.

Recent advances in computational drug discovery have introduced integrated approaches combining machine learning (ML), molecular docking, molecular dynamics (MD) simulations, and free-energy calculations for the efficient identification of bioactive compounds from natural product libraries [38–41]. ML algorithms facilitate rapid screening and prioritization of potential inhibitors from large phytochemical datasets, while molecular simulations provide detailed insights into protein–ligand interactions, conformational stability, and binding mechanisms [41,42]. Among the advanced simulation techniques, umbrella sampling (US) offers reliable estimation of ligand-binding free energies and dissociation pathways under dynamic physiological conditions [43,44].

Therefore, the present study aimed to identify potential ALK inhibitors from *Glycosmis*-derived phytochemicals using an integrated computational workflow involving ML prediction, molecular docking, MD simulation, and US analyses (supplementary material, figure S1). Systematic exploration of the phytochemical diversity within *Glycosmis* species may provide valuable insights into novel natural-product-derived ALK inhibitors and contribute to the future development of targeted anticancer therapeutics.

## 2. Material and methods

### 2.1. Compound structure preparation

The phytochemical constituents of *Glycosmis* were collected from previously published studies on the isolation and characterization of compounds from species within this genus [1,45,46]. All chemical structures were constructed using ChemSketch software and subsequently optimized through molecular mechanics energy minimization with the MMFF94s force field implemented in Avogadro2, yielding stable and energetically favorable molecular geometries [47,48]. The optimized structures were then converted into the SMILES format using Open Babel for subsequent ML applications [49]. In addition, ligand structures were prepared in the PDBQT format using AutoDockTools to facilitate molecular docking studies.

### 2.2. CatBoost-based machine learning approach for rapid virtual screening

The ML workflow was constructed based on previously published studies aimed at rapid virtual screening and structure–activity relationship (SAR) analysis of phytochemical compounds derived from *Glycosmis* species [50]. As the first step, canonical SMILES strings of phytochemical compounds were prepared as molecular input data for computational analysis. From these SMILES strings, a comprehensive set of molecular descriptors, structural fingerprints and physico-chemical properties were systematically generated and subsequently employed as feature inputs for ML modelling. To establish a predictive model for the ALK target, a dataset comprising 3169 experimentally validated ALK inhibitors was retrieved from the ChEMBL database. The canonical SMILES strings of these inhibitors were initially converted into 217 RDKit molecular descriptors, of which 145 were retained following data preprocessing and feature filtering. The resulting dataset was subsequently divided into training and test sets at an 80 : 20 ratio. Model hyperparameters were optimized using Hyperopt, while 10-fold cross-validation was employed to evaluate model robustness and minimize the risk of overfitting. Among the evaluated algorithms, the CatBoost algorithm was chosen to predict the binding free energy of *Glycosmis*-derived compounds against the ALK protein target owing to its robust performance in capturing complex nonlinear relationships and its effectiveness in handling high-dimensional, heterogeneous molecular feature spaces. The optimized model achieved a root mean square error (RMSE) of 0.933, a mean absolute error (MAE) of 0.719 and a Pearson correlation coefficient of 0.808. This ML framework enables the efficient extraction and integration of structural and physico-chemical information from phytochemical datasets, thereby facilitating the rapid prioritization of potentially bioactive molecules.

### 2.3. Preparation of ALK protein structures and co-crystallized ligands

The three-dimensional structures of ALK and its co-crystallized inhibitors were retrieved from the Protein Data Bank (PDB), comprising the following entries: 2XP2, 3AOX, 4CCB, 4CCU, 4CMO, 4CTB, 4CTC, 4CNH, 4MKC, 5AAA, 5AAB and 5AAC [51–56]. All protein structures were prepared, including the removal of all crystallographic water molecules and ligands, followed by the addition of polar hydrogen atoms using the AutoDockTools software. Although structural waters may mediate ligand binding, they were removed to ensure consistent receptor preparation and focus on direct protein–ligand interactions. The co-crystallized ligand structures were separately extracted and saved in the PDB format using PyMOL software. The prepared structures were subsequently used for protocol validation and downstream molecular modelling studies.

### 2.4. Molecular docking

Based on the predictions obtained from the ML model, the compounds with the most favourable predicted binding free energy were selected for further molecular docking analysis to enable additional screening and formation of ligand–protein complexes with the target protein ALK. Molecular docking was performed using AutoDock Vina (version 1.2.7) [57,58]. Among the evaluated ALK crystal structures, the structure with PDB ID 5AAB was selected as the receptor for docking of the ML-prioritized compounds. The three-dimensional structures of ALK were pre-processed, followed by assignment of Kollman charges to the protein. The selected ligands were prepared by assignment of Gasteiger charges and the definition of rotatable bonds, and both protein and ligand files were converted into the PDBQT format. The docking grid box was centred on the active site of ALK, defined based on the position of co-crystallized ligands from experimentally resolved structures, ensuring complete coverage of key catalytic residues within the binding pocket. The grid box dimensions were carefully adjusted to accommodate ligand flexibility while maintaining computational efficiency. For this virtual screening stage, the same docking search space was consistently applied to all selected compounds. The grid was centred on the ligand-binding pocket at *x* = 29.6 Å, *y* = 48.1 Å and *z* = 8.8 Å, with box dimensions of 24 × 24 × 24 Å. Docking simulations were conducted using an exhaustiveness parameter of 400 to ensure reliable sampling of ligand conformations. For each ligand, multiple binding poses were generated, with the conformation exhibiting the lowest binding free energy (kcal mol^−1^) selected as the most favourable binding mode.

### 2.5. Molecular dynamics simulations

GROMACS v. 2024.1 was employed to perform atomistic MD simulations [59]. Selected conformations of the docked complexes were used as initial structures for MD simulations. The molecular system, including the protein with bound ions, ligand and explicit solvent molecules, was parametrized using the established force fields. Specifically, the protein and ions were described using the Amber99SB-ILDN force field, the ligand was treated with the general Amber force field (GAFF), and water molecules were modelled using the TIP3P water model [60–62]. Counterions were initially added to neutralize the net charge of each system. All MD simulations were carried out following protocols that were consistent with previous studies [50,63]. The integration time step was set as 2 fs. A cut-off distance of 1.2 nm was applied for short-range non-bonded interactions. Long-range electrostatic interactions were evaluated using the particle mesh Ewald (PME) method [64], whereas van der Waals interactions were handled using a standard cut-off scheme. All covalent bonds involving hydrogen atoms were constrained using the LINCS algorithm. Before the production runs, the solvated protein–ligand complexes were subjected to energy minimization, followed by number of particles, volume and temperature (NVT) equilibration for 500 ps at 310 K using the V-rescale thermostat. Subsequently, number of particles, pressure and temperature (NPT) equilibration was performed for 1000 ps at 1 bar using the Parrinello–Rahman barostat. After equilibration, 200 ns production MD simulations were carried out with a 2 fs integration time step. Trajectory coordinates were saved every 10 ps for subsequent analysis. Finally, MD simulations were performed for 200 ns to investigate the structural stability and dynamic behaviour of the complexes over time.

### 2.6. Steered molecular dynamics

The equilibrated MD structure of the protein–ligand complex was selected as the initial configuration for steered MD (SMD) simulations. In each SMD run, the ligand was gradually extracted from the binding pocket by applying an external harmonic force acting on the centre of mass along a defined reaction coordinate (*z*-axis). A spring constant of *k* = 600 kJ mol⁻¹ nm⁻² was used to define the strength of the restraining force, while the pulling speed was maintained at 0.005 nm ps⁻¹. Each trajectory was propagated for 0.5 ns, in agreement with previously established SMD protocols, to ensure methodological consistency and reliable comparison of results [39,50]. During the pulling process, key dynamical quantities, including the reaction coordinate, applied force and accumulated work, were sampled at 0.1 ps intervals for further post-processing and analysis.

### 2.7. Umbrella sampling simulations

US simulations were constructed based on a selected SMD pathway, in which the trajectory exhibiting the smallest deviation in pulling work from the ensemble average was chosen as the most representative dissociation route. From this optimized pathway, a series of initial configurations were extracted to define US windows along the reaction coordinate, with an average spacing of approximately 0.05 nm between neighbouring states. Approximately 50 windows were generated to ensure sufficient coverage of the ligand-unbinding process. For each window, the system was first subjected to a short equilibration phase (0.1 ns) under the NPT conditions to relax any structural artefacts inherited from the SMD pulling procedure. This was followed by a 2 ns production simulation to obtain adequate sampling of each restrained state. To enhance the computational efficiency and maintain structural stability, weak harmonic positional restraints were applied to the Cα atoms of the protein through both SMD and US simulations. This strategy prevents large-scale translational and rotational motions of the protein while preserving its local conformational flexibility, thereby avoiding the need for exhaustive sampling of the full protein conformational space. Such an approximation is reasonable, given the limited structural changes in the protein backbone upon ligand binding. Finally, the potential mean force (PMF) was reconstructed from all umbrella windows using standard statistical reweighting analysis [65,66]. Statistical uncertainty in the PMF profile was evaluated using bootstrap analysis.

### 2.8. Toxicity prediction

The toxicity profiles of the investigated compounds were evaluated using ProTox 3.0, an online platform that combines ML approaches with curated and experimentally validated toxicological data [67]. This tool enables the prediction of acute oral toxicity expressed as the median lethal dose (LD₅₀, mg kg⁻¹ body weight), along with classification according to the Globally Harmonized System of Classification and Labelling of Chemicals (GHS). Based on this system, compounds are categorized into six toxicity classes, ranging from class 1 (highly toxic) to class 6 (practically non-toxic). In addition to LD₅₀ estimation and GHS classification, ProTox 3.0 provides predictions of organ-specific toxicities, including hepatotoxicity, neurotoxicity, nephrotoxicity, respiratory toxicity and cardiotoxicity. These toxicological endpoints are reported qualitatively as either ‘Active’ or ‘Inactive’, depending on probability thresholds derived from the underlying predictive models. All compounds were submitted to the platform in SMILES format, and the resulting toxicity parameters were collected for subsequent analysis and comparative evaluation.

## 3. Results and discussion

### 3.1. Machine learning–based prediction of binding free energy

ML has emerged as a powerful and efficient approach in modern drug discovery, enabling rapid screening and prioritization of large chemical libraries based on predicted bioactivity and physico-chemical properties. By learning complex nonlinear relationships between molecular descriptors and biological responses, ML models can significantly reduce the time and cost associated with traditional experimental and computational methods [39,63,68,69]. In particular, ensemble learning algorithms, such as CatBoost, have demonstrated high predictive accuracy, robustness to overfitting and the ability to effectively handle complex and heterogeneous chemical datasets.

In this study, multiple ML algorithms, including linear regression (LR), K-nearest neighbours (KNN), support vector regression (SVR), random forest (RF), gradient boosting (GB), XGBoost, LightGBM and CatBoost, were previously evaluated for predicting the binding free energy of the ALK target. In a previous study, the CatBoost model achieved the highest correlation coefficient (R) among the tested models, indicating its superior predictive capability and reliability [50]. This validation provides a strong rationale for selecting CatBoost as the optimal model in the present study.

Accordingly, the optimized CatBoost model was employed to predict the binding free energy (Δ*G*_ML_) for a dataset of 307 phytochemical compounds derived from *Glycosmis*. The prediction results are summarized in figure 1 and supplementary material, table S1. As illustrated in figure 1, the Δ*G*_ML_ values range from −8.27758 to −11.4927 kcal mol^−1^, with a median value of −10.1833 kcal mol^−1^, indicating an overall favourable binding tendency of these natural compounds toward the target protein. Notably, 26 compounds with Δ*G*_ML_ values lower than −11 kcal mol^−1^ were selected for further investigation, representing the most promising candidates based on the ML predictions. This threshold was chosen to prioritize high-affinity compounds that outperformed the median and fell within the most favourable range of the distribution. These candidates are more likely to form stable protein–ligand complexes and act as potent inhibitors. Selecting the 26 top-ranked compounds also balances computational efficiency with sufficient chemical diversity for subsequent *in silico* analyses. The selected compounds were subsequently subjected to advance *in silico* evaluations, including MD simulations and rigorous binding free energy calculations. These approaches provide deeper insights into the stability, conformational flexibility and key intermolecular interactions governing ligand binding at the molecular level, thereby complementing initial ML-based screening and enhancing the reliability of the overall drug discovery pipeline. It should be noted that ML, molecular docking, MD and US provide complementary levels of evaluation within a progressive prioritization workflow. Since these approaches assess different aspects of ligand–protein interactions, changes in compound ranking between stages are expected.

**Figure 1.**
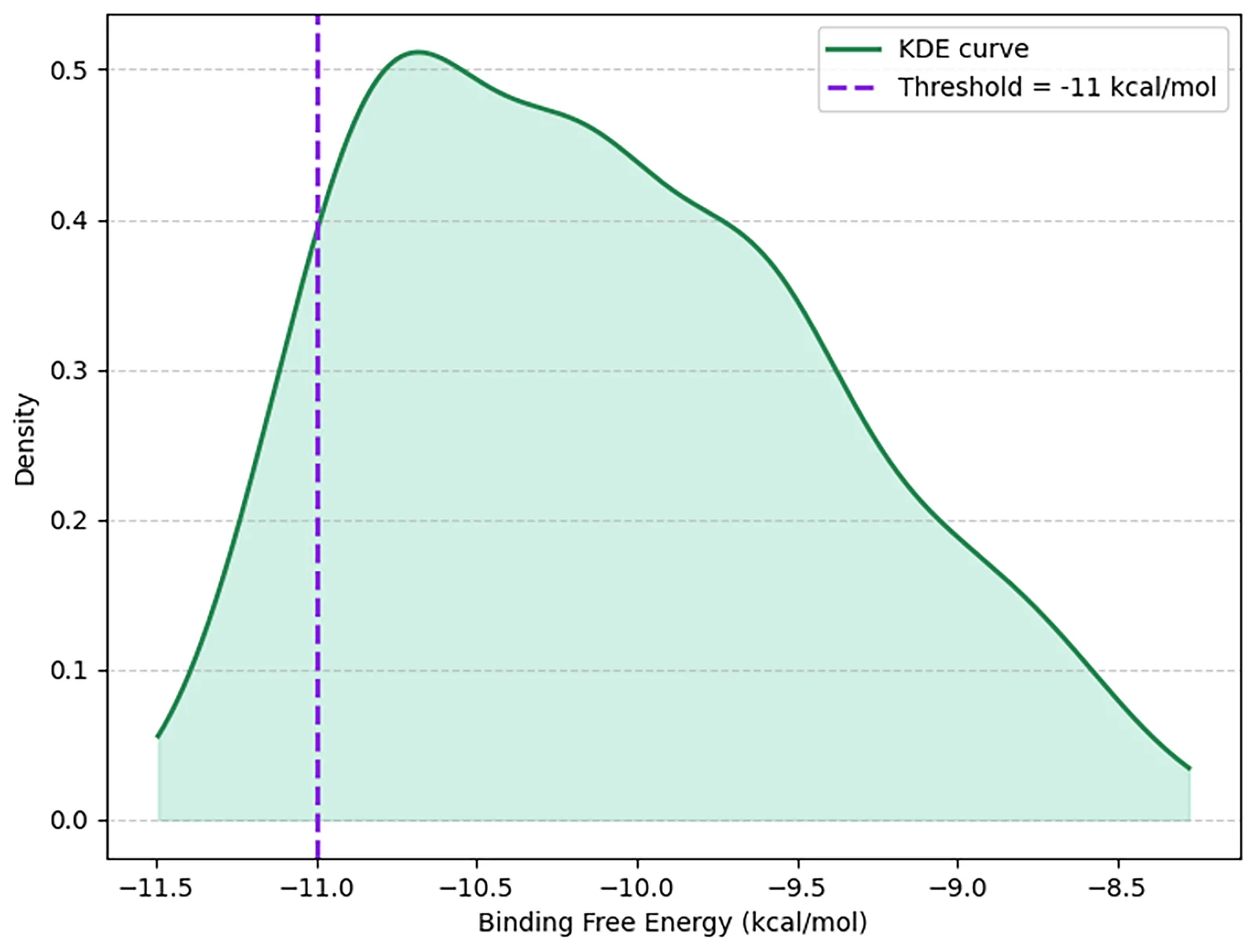
Kernel density estimation (KDE) plot of predicted binding energies for *Glycosmis* phytochemicals against the ALK protein using the CatBoost model.

### 3.2. Molecular simulations

A total of 26 compounds were further evaluated using molecular simulations targeting the ALK protein. Initially, molecular docking was performed to narrow down the most promising compounds predicted by the ML model, while simultaneously generating the corresponding protein–ligand complexes. Before virtual screening, the docking protocol was validated by redocking the co-crystallized ligands into 12 ALK crystal structures. The root mean square deviation (RMSD) values between the redocked and crystallographic poses ranged from 0.253 to 1.695 Å (supplementary material, table S2), with all values below the commonly accepted threshold of 2.0 Å, confirming that the docking protocol could reliably reproduce the experimentally observed binding modes. The docking results are summarized in supplementary material, table S3 and figure 2. The calculated binding affinities (Δ*G*_dock_) ranged from −5.856 to −9.667 kcal mol^−1^, indicating favourable interactions with the ALK active site. Among these, six compounds (figure 3) with binding energies lower than −9 kcal mol^−1^ were selected for subsequent MD simulations. The selected ALK–ligand complexes were subjected to independent 200 ns MD simulations to assess their stability and dynamic behaviour. The backbone RMSD profiles (figure 4A) demonstrated that all systems reached equilibrium during the simulation, with average RMSD values ranging from 0.154955 to 0.181893 nm, consistently remaining below 3 Å, which indicates good structural stability of the protein. Similarly, ligand RMSD analysis (figure 4B) revealed that most compounds remained stable within the ALK-binding pocket, with average values ranging from 0.057259 to 0.167617 nm. Notably, compound 54 exhibited an initial increase in RMSD during the first 50 ns, followed by stabilization for the remainder of the simulation. Importantly, all ligand RMSD values remained below 2 Å, confirming that the ligands were well maintained within the active site and formed stable complexes throughout the simulation period. In addition, the radius of gyration (Rg) (figure 4C) was analysed to evaluate the compactness and overall structural integrity of the protein during the simulations. The Rg values remained stable within a narrow range of 2.00155–2.02462 nm, suggesting that no significant structural expansion or collapse occurred. This consistency further supports the conformational stability of the ALK protein upon ligand binding and indicates that the complexes maintained a well-folded and compact structure throughout the simulation.

**Figure 2.**
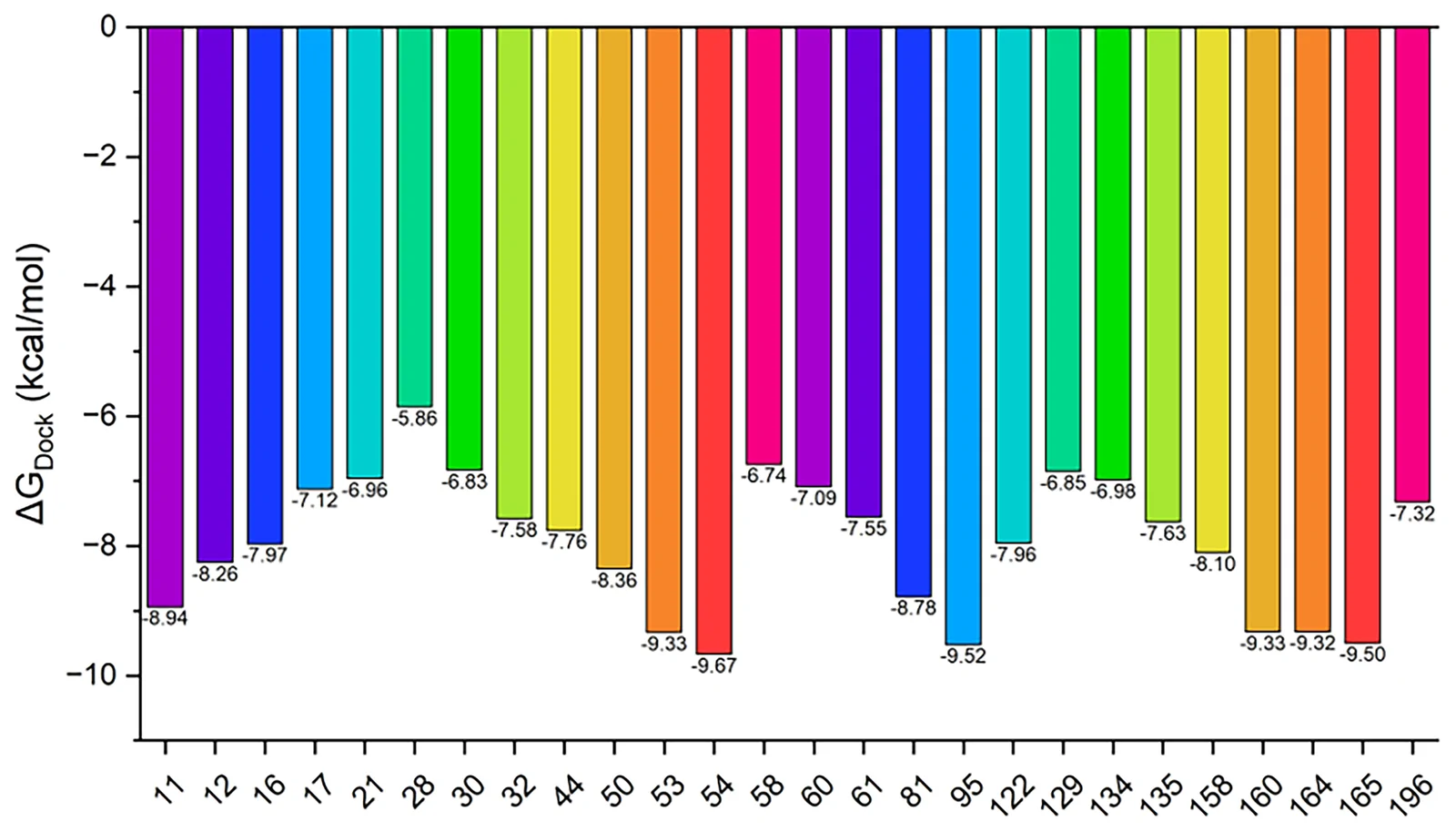
Binding energies of selected compounds toward the ALK protein predicted by molecular docking.

**Figure 3.**
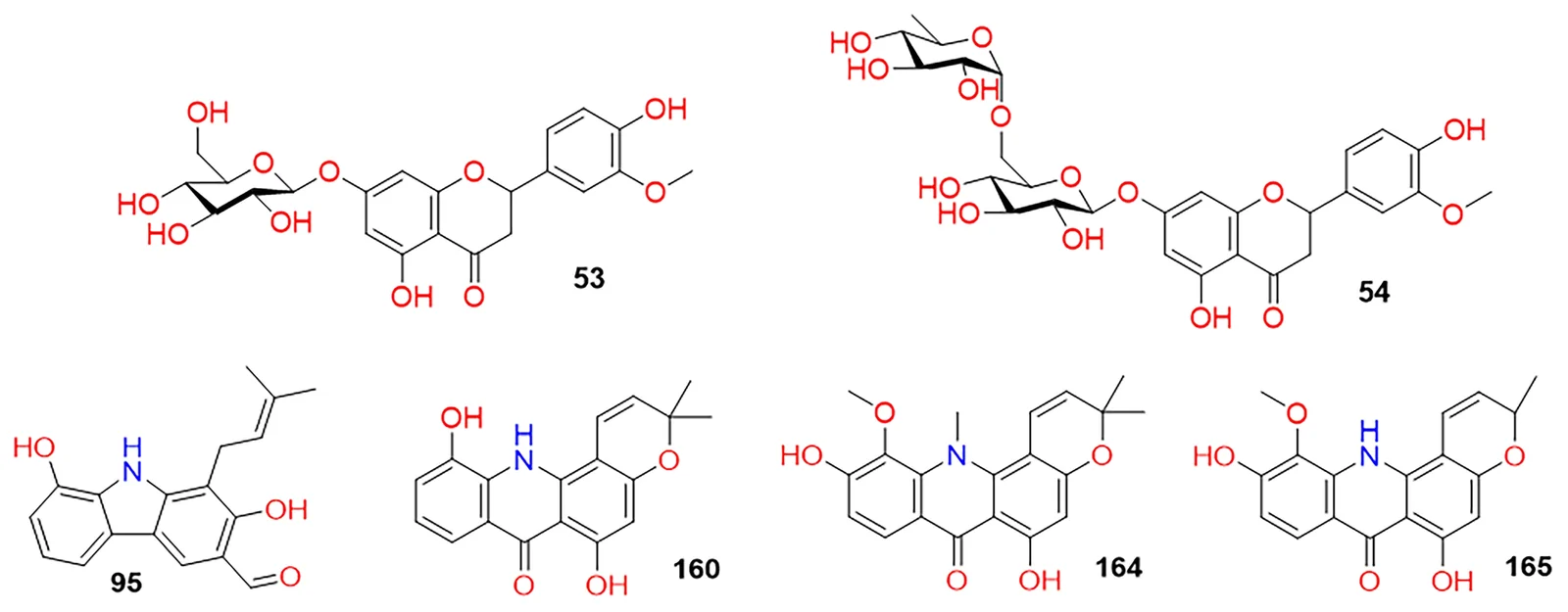
Chemical structures of the top potential ALK inhibitors.

**Figure 4.**
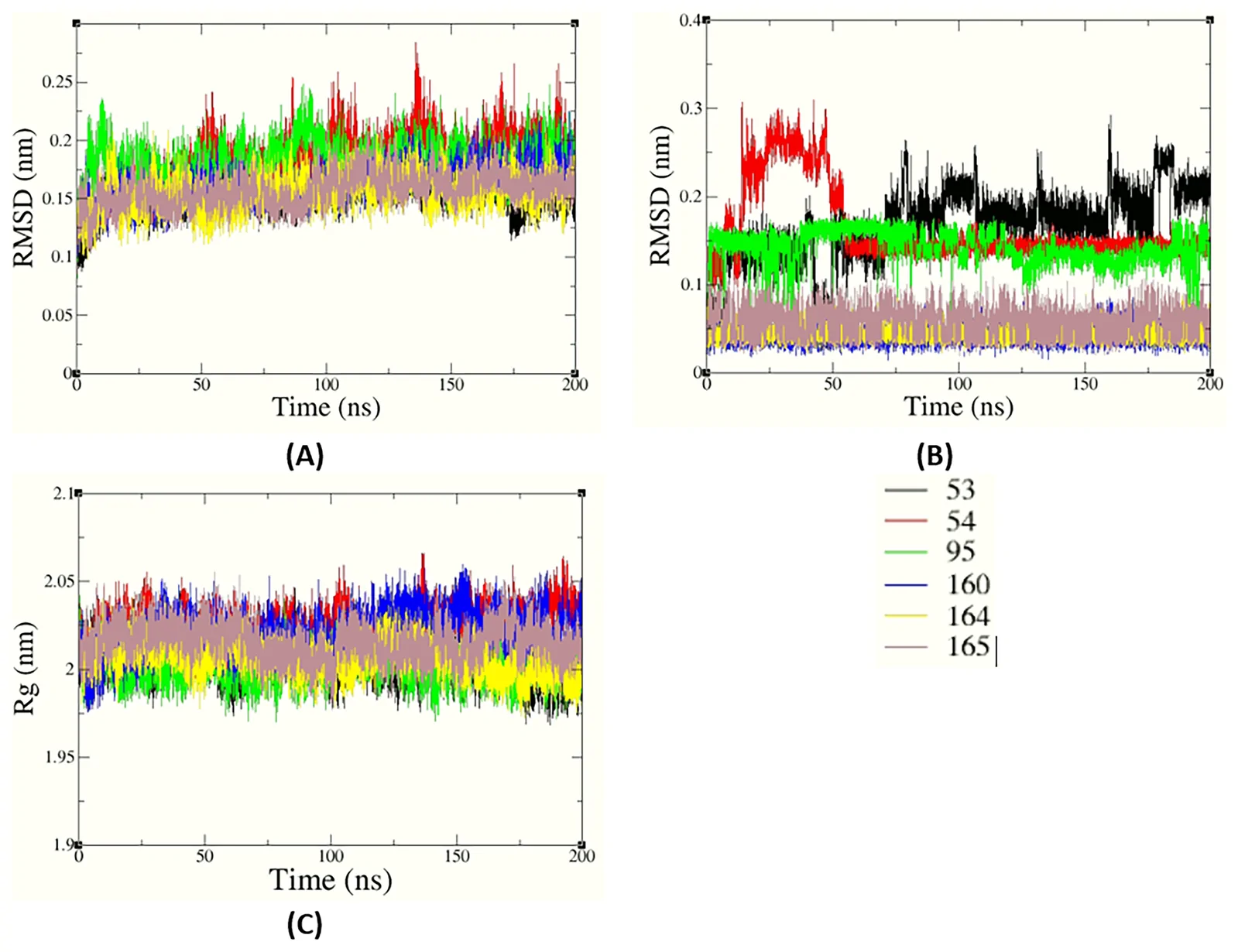
Time-dependent evaluation of backbone RMSD (A), ligand RMSD (B) and radius of gyration (Rg) (C) revealing the dynamic stability of ALK protein–ligand complexes over 200 ns.

Furthermore, free-energy landscape (FEL) analysis was performed to investigate the conformational variations of the ALK backbone induced by ligand binding. Approximately 2000 snapshots extracted from the 200 ns MD trajectories for each system were clustered based on RMSD and Rg. The resulting FEL profiles (figure 5) revealed distinct conformational basins corresponding to energetically favourable states sampled during the simulations. In all systems, a dominant deep-energy minimum was observed, indicating that the complexes predominantly occupied stable conformational states with limited structural fluctuations throughout the simulation period. The presence of a single major energy basin suggests that ligand binding did not induce significant destabilization or large-scale conformational rearrangements within the ALK catalytic domain.

**Figure 5.**
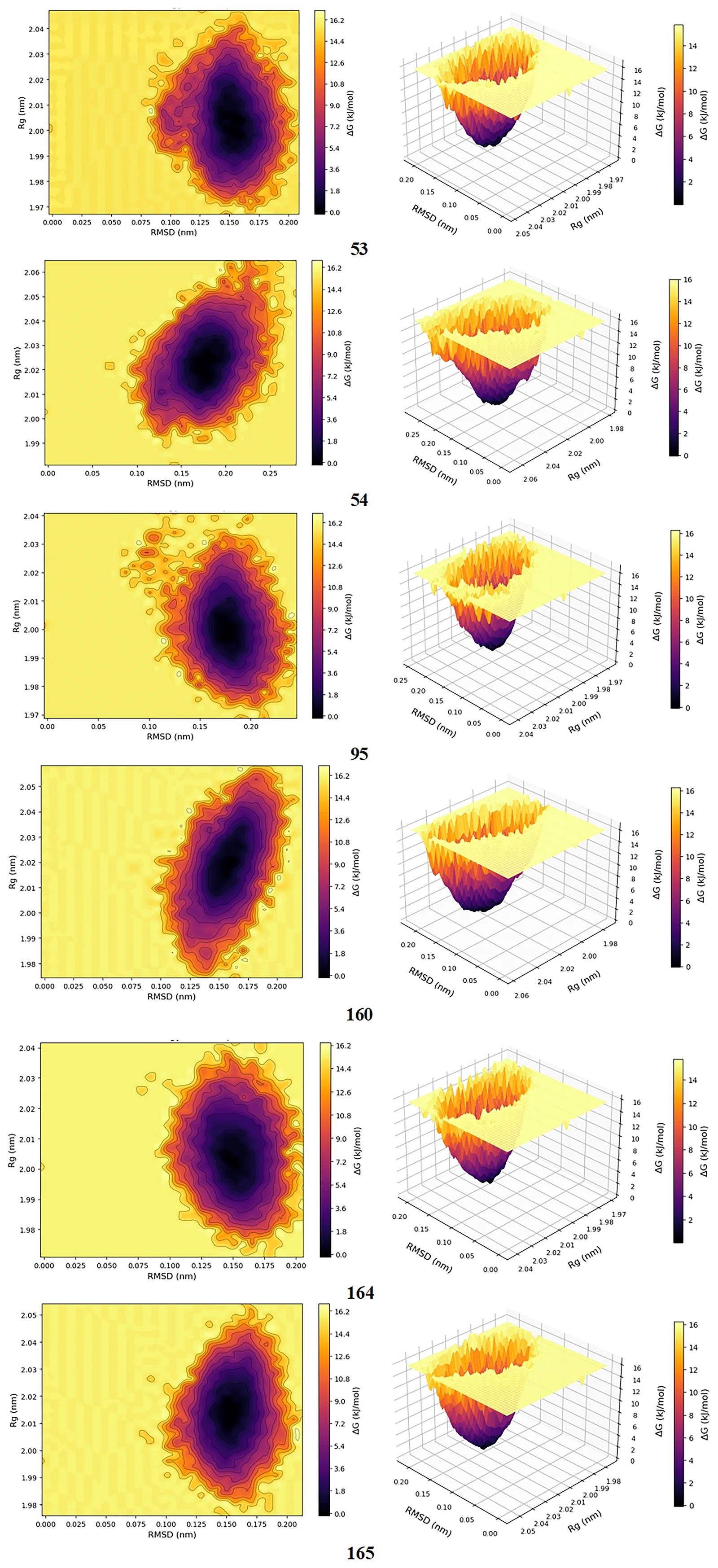
Two-dimensional and three-dimensional free energy landscapes of the protein–ligand complex derived from RMSD and radius of gyration (Rg) during 200 ns md simulations.

The representative conformations extracted from the global minimum regions of the FEL were subsequently used for protein–ligand interaction analysis. The detailed interaction mapping is shown in figures 6 and 7. The hydrophobic contacts of all selected compounds within the ALK active pocket were mainly formed with several key residues, including Leu1122, Ala1148, Val1130, Leu1256, Gly1202 and Met1199, which were repeatedly involved in stabilizing ligand binding through hydrophobic interactions. These residues have also been frequently reported in the binding patterns of co-crystallized ALK inhibitors, highlighting their critical role in maintaining inhibitor affinity within the ATP-binding cavity [70,71].

**Figure 6.**
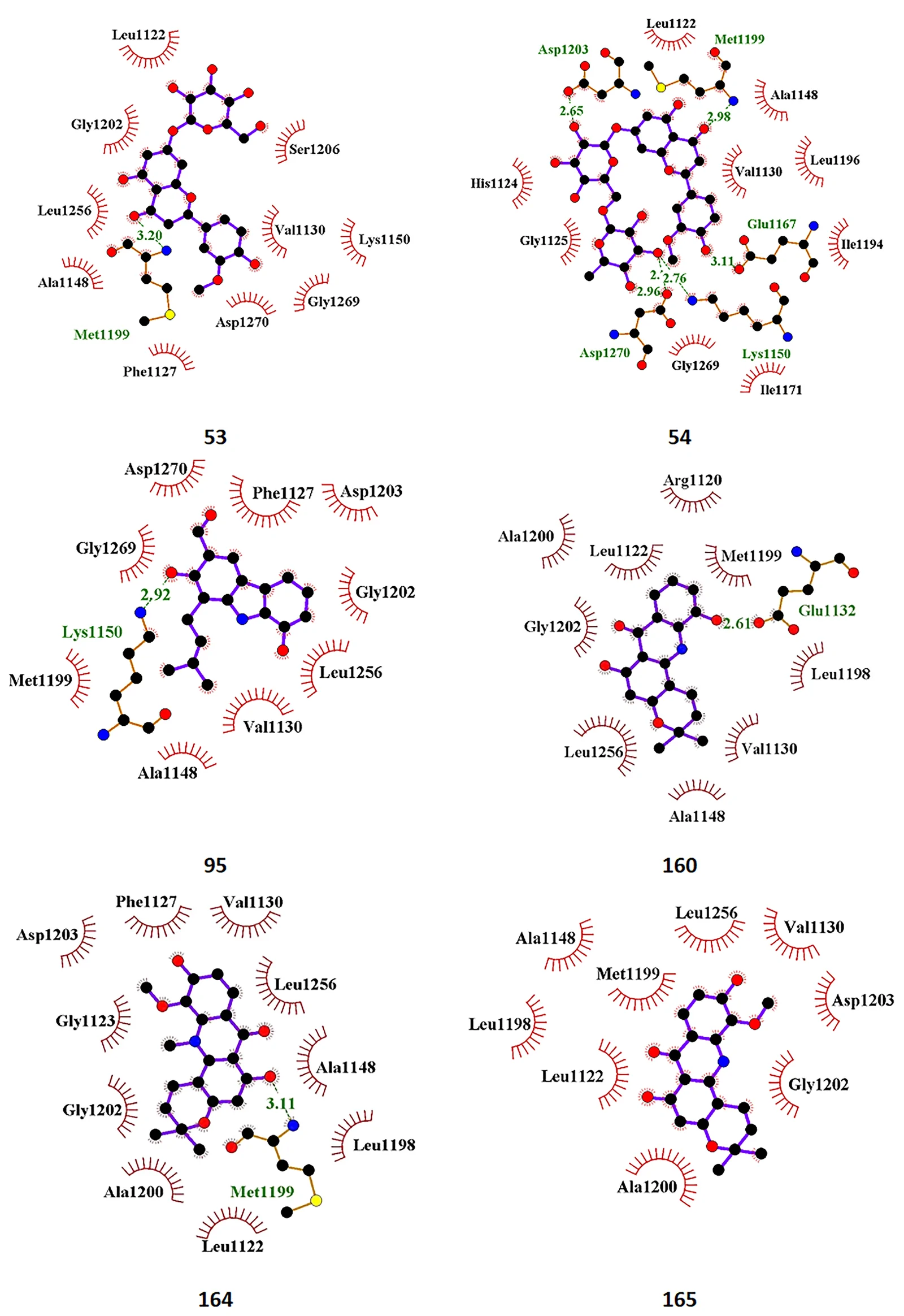
Interaction profiles of representative conformations selected from the free energy landscape (FEL) of each studied system.

**Figure 7.**
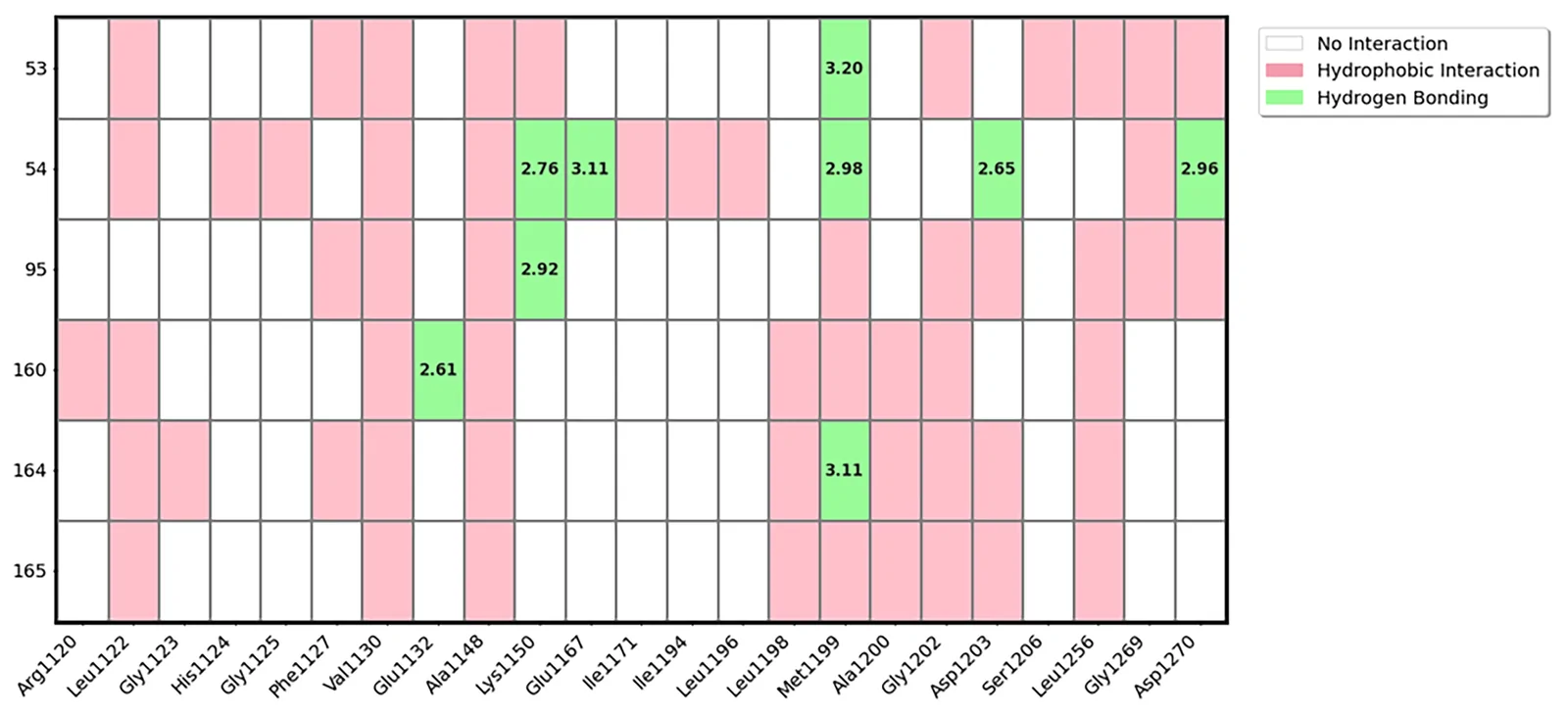
Heatmap representation of hydrophobic and hydrogen bond interactions between selected ligands and key amino acid residues within the active site of ALK protein.

In addition to hydrophobic contacts, hydrogen bonding interactions significantly contribute to the binding affinity. Compound 54 formed the most hydrogen bonds with Asp1203, Met1199, Glu1167, Lys1150 and Asp1270, with interaction distances ranging from 2.65 to 3.11 Å, indicating strong and stable intermolecular interactions. Compound 53 established a hydrogen bond with Met1199 at a distance of 3.2 Å, whereas compound 95 interacted with Lys1150 through a hydrogen bond distance of 2.92 Å. Compound 160 formed a particularly strong hydrogen bond with Glu1132 at 2.61 Å, suggesting enhanced electrostatic stabilization within the binding pocket. Compound 164 also maintained a hydrogen bond with Met1199 at 3.11 Å, whereas compound 165 primarily relied on extensive hydrophobic interactions, without forming significant hydrogen bonds.

Comparison with reported co-crystallized ALK inhibitors revealed that the selected compounds shared several key binding characteristics with known co-crystallized inhibitors, particularly through extensive hydrophobic contacts and conserved hydrogen-bonding interactions within the active pocket. Most reference inhibitors consistently interacted with key residues, such as Met1199, Glu1197, Leu1122, Ala1148, Gly1202 and Leu1256. Similarly, compounds 53, 54, 95, 160, 164 and 165 occupied the same hydrophobic cavity and established interactions with many of these crucial residues, indicating a comparable binding orientation within the active site. Overall, the identified compounds demonstrated interaction patterns that were highly comparable to those of the established inhibitors, particularly through conserved contacts with Met1199, Leu1122, Ala1148, Leu1256, Gly1202 and Val1130. These similarities suggested that the investigated compounds may adopt analogous binding conformations and possess promising inhibitory potential against the target protein.

### 3.3. Binding free energy between promising candidate compounds and ALK through umbrella sampling simulations

To validate the reliability of the US protocol for estimating binding free energy, the binding affinities of 12 experimentally characterized ALK–ligand complexes, for which both crystal structures and half-maximal inhibitory concentration (IC₅₀) data were available, were initially calculated. As summarized in supplementary material, table S3, the calculated values were in good agreement with the experimental data. The PMF profiles along the reaction coordinate (ξ) for the co-crystallized ligands in complexes with PDB IDs 2XP2, 3AOX, 4CTC, 4CNH, 4MKC, 5AAA, 5AAB, 4CCU, 4CTB, 4CCB, 4CMO and 5AAC, obtained from US simulations are presented in supplementary material, figure S2. In particular, correlation analysis between the calculated and experimental binding free energies (figure 8) yielded a Pearson correlation coefficient of *R* = 0.834, indicating that the US protocol could reliably estimate the binding free energies of ALK–ligand complexes, in agreement with experimental observations. Therefore, this validated protocol was subsequently applied to six top-ranked candidate compounds; the resulting binding free energies are presented in table 1.

**Figure 8.**
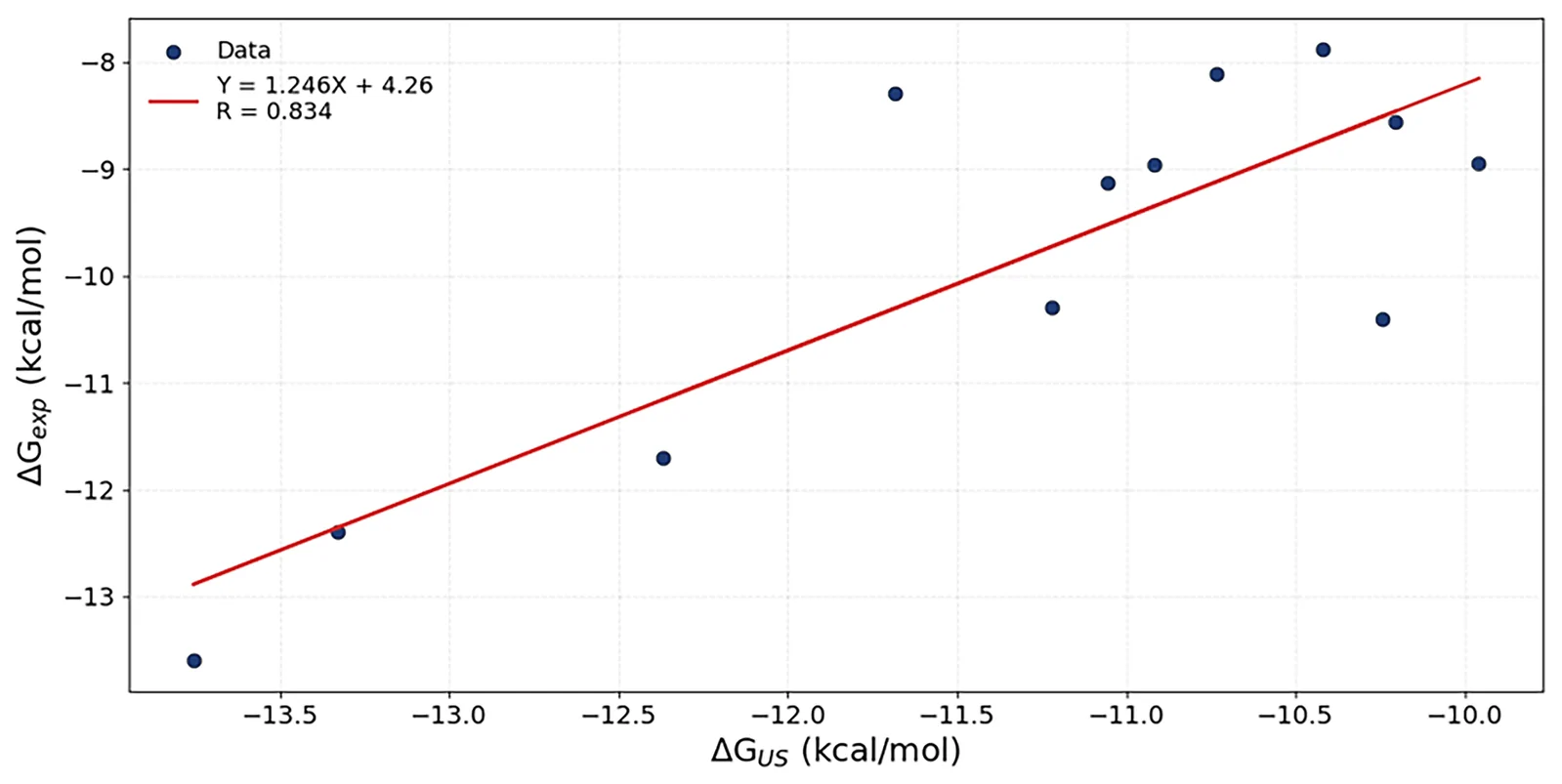
Correlation analysis between predicted binding free energies from PMF calculations (Δ*G*_US_) and experimental binding energies (Δ*G*_exp_) of ALK inhibitors.

**Table 1.** Docking, MD, SMD and US results of selected compounds with ALK protein.

ID	compound name	Δ*G*_Dock_ (kcal mol^−1^)	*F*_max_ (pN)	RMSD of backbone (nm)	RMSD of ligand (nm)	Rg (nm)	Δ*G*_US_ (kcal mol^−1^)
**53**	5,4′-dihydroxy-3′-methoxy-7-O-β-D-glucupyranosyl flavanone	−9.334	703.184	0.155036	0.167617	2.00496	−9.796
**54**	5,4′-dihydroxy-3′-methoxy-7-*O*-(*α*-L-rhamnosyl-(1‴→6‴)-β-D-glucopyranosyl) flavanone	−9.667	1260.13	0.181721	0.162788	2.02462	−13.272
**95**	heptazoline	−9.523	634.069	0.181893	0.142593	2.00155	−8.716
**160**	atalaphyllidine	−9.327	479.388	0.161271	0.040608	2.02035	−8.354
**164**	citracridone-I	−9.324	662.229	0.154955	0.043725	2.00572	−8.595
**165**	acrifoline	−9.499	466.468	0.157299	0.057259	2.01527	−10.317

Representative conformations extracted from the 200 ns MD simulations were subsequently subjected to SMD simulations (supplementary material, figure S3), followed by US calculations to estimate the binding free energies (Δ*G*_US_) of the selected compounds toward ALK. The predicted Δ*G*_US_ values ranged from −8.354 to −13.272 kcal mol^−1^ (table 1; supplementary material, figure S4). Among the investigated compounds, compound 54, identified as the flavonoid 5,4′-dihydroxy-3′-methoxy-7-O-(α-L-rhamnosyl-(1‴→6‴)-*β*-D-glucopyranosyl) flavanone, exhibited the most favourable binding free energy with a Δ*G*_US_ value of −13.272 kcal mol^−1^. This compound was followed by compound 165, the alkaloid acrifoline (Δ*G*_US_ = −10.317 kcal mol^−1^); compound 53, 5,4′-dihydroxy-3′-methoxy-7-*O*-*β*-D-glucopyranosyl flavanone (Δ*G*_US_ = −9.796 kcal mol^−1^); compound 160, the alkaloid heptazoline (Δ*G*_US_ = −8.716 kcal mol^−1^); compound 95, citracridone-I (Δ*G*_US_ = −8.595 kcal mol^−1^); and compound 164, atalaphyllidine (Δ*G*_US_ = −8.354 kcal mol^−1^). Notably, several of the identified alkaloids have previously demonstrated promising biological activities in experimental studies. Acrifoline showed potent inhibitory activity against dual-specificity tyrosine phosphorylation-regulated kinase 1A (DYRK1A) with an IC₅₀ value of 0.075 µM, while atalaphyllidine displayed considerable inhibition with an IC₅₀ value of 2.2 µM [9]. Furthermore, heptazoline exhibited significant anticancer activity against SCC-15 oral cancer cells with an IC₅₀ value of 12 µM, while showing substantially lower cytotoxicity toward normal hTRET-OME cells (IC₅₀ = 85 µM) [72]. In addition, heptazoline showed strong cytotoxicity against NCI-H187 cells with an IC₅₀ value of 1.63 µg ml^−1^, further supporting its anticancer potential [73]. Mechanistic investigations revealed that heptazoline induced apoptosis through DNA damage, Bax/Bcl-2 regulation, caspase-3 activation and suppression of the PI3K/AKT signalling pathway [72]. In addition, atalaphyllidine exhibited considerable antiproliferative activity against multiple cancer cell lines, including A-549 lung carcinoma, B-16 melanoma 4A5, CCRF-HSB-2 leukaemia and TGBC-11TKB cells, with IC₅₀ values of 6.98, 5.58, 4.65 and 2.83 µM, respectively [74]. Collectively, the favourable binding free energies predicted by US, together with previously reported experimental anticancer and kinase inhibitory activities, strongly support the therapeutic potential of these natural compounds as promising potential ALK inhibitors. These compounds emerged as promising candidates for further experimental validation and optimization.

### 3.4. Predicted toxicity profiles of selected compounds

Early toxicity assessment is a crucial step in the drug discovery and development process, particularly for natural compounds intended for long-term therapeutic applications. Although many phytochemicals exhibit promising biological activities, unfavourable toxicity profiles may limit their pharmaceutical potential, lead to late-stage clinical failure, and cause severe adverse effects *in vivo*. Therefore, evaluating acute toxicity and organ-specific toxicological risks at the early stages of computational screening helps prioritize safer candidates, reduce development costs and improve the probability of successful drug development. In this context, *in silico* toxicity prediction provides a rapid and cost-effective approach to estimate the safety profiles of bioactive compounds before experimental validation.

The toxicity prediction results obtained from ProTox 3.0 revealed that the selected compounds exhibited generally low to moderate acute toxicity profiles, as shown in table 2. Specifically, the predicted oral LD₅₀ values ranged from 522 to 12 000 mg kg^−1^, corresponding to toxicity classes 4–6 under the Globally Harmonized System (GHS). Among them, compound 54 showed the highest safety margin with an LD_50_ of 12 000 mg kg^−1^ (class 6), indicating that it is relatively non-toxic, whereas compound 160 exhibited the lowest LD_50_ value (522 mg kg^−1^, class 4), suggesting comparatively higher toxicity but still within an acceptable range for drug candidates.

**Table 2.** Predicted acute toxicity (LD_50_), toxicity classes and target organ toxicities of selected compounds obtained from the protox 3.0 web server.

ID	predicted LD_50_	predicted toxicity class	hepato.	neuro.	nephro.	respi.	cardio.
**53**	2300	5	inactive (*p* = 0.83)	inactive (*p* = 0.86)	active (*p* = 0.76)	active (*p* = 0.61)	inactive (*p* = 0.63)
**54**	12 000	6	inactive (*p* = 0.81)	inactive (*p* = 0.87)	active (*p* = 0.76)	active (*p* = 0.66)	inactive (*p* = 0.53)
**95**	1000	4	inactive (*p* = 0.59)	inactive (*p* = 0.73)	inactive (*p* = 0.54)	active (*p* = 0.73)	inactive (*p* = 0.82)
**160**	522	4	inactive (*p* = 0.68)	inactive (*p* = 0.75)	inactive (*p* = 0.66)	active (*p* = 0.70)	inactive (*p* = 0.79)
**164**	1000	4	inactive (*p* = 0.67)	inactive (*p* = 0.51)	inactive (*p* = 0.67)	active (*p* = 0.74)	inactive (*p* = 0.90)
**165**	1000	4	inactive (*p* = 0.65)	inactive (*p* = 0.69)	inactive (*p* = 0.60)	active (*p* = 0.71)	inactive (*p* = 0.77)

*Note*s: hepato, hepatotoxicity; neuro, neurotoxicity; nephro, nephrotoxicity; respi, respiratory toxicity; cardio, cardiotoxicity.

Regarding organ-specific toxicity, most compounds were predicted to be inactive against hepatotoxicity and neurotoxicity, with high confidence probabilities (*p* > 0.5), indicating a low risk of liver and nervous system damage. Similarly, nephrotoxicity and cardiotoxicity were consistently predicted to be inactive across all compounds, suggesting favourable safety profiles for kidney and heart function. However, respiratory toxicity emerged as a common concern, as all compounds were predicted to be active with moderate probabilities (*p* ≈ 0.61–0.74), which may indicate a potential risk of respiratory system effects and should be carefully considered in further *in vitro* and *in vivo* evaluations. These findings suggest that the investigated compounds possessed acceptable toxicity characteristics, particularly in terms of acute toxicity and major organ safety. Nonetheless, the predicted respiratory toxicity highlights the need for additional experimental validation to confirm the safety profiles before further pharmaceutical development.

## 4. Conclusion

The present study successfully integrated ML, molecular docking, MD simulations, US and toxicity prediction to identify potential ALK inhibitors from *Glycosmis* phytochemicals. The previously validated CatBoost model enabled the rapid and reliable screening of 307 natural compounds, leading to the identification of 26 promising candidates with highly favourable predicted binding free energies. Subsequent molecular docking and 200 ns MD simulations demonstrated that the selected compounds formed stable ALK–ligand complexes and maintained favourable interactions within the ATP-binding pocket throughout the simulations. Interaction analyses further revealed conserved hydrophobic and hydrogen-bonding interactions with key catalytic residues, including Met1199, Leu1122, Ala1148, Gly1202 and Leu1256, which are commonly associated with potent ALK inhibition. Among the investigated compounds, 54 exhibited the most favourable US binding free energy, followed by several alkaloids, including acrifoline, heptazoline, citracridone-I and atalaphyllidine, which also demonstrated strong binding affinities and previously reported anticancer or kinase inhibitory activities. In addition, the predicted toxicity profiles suggested generally acceptable safety characteristics, with low to moderate acute toxicity and minimal risks of hepatotoxicity, nephrotoxicity, neurotoxicity and cardiotoxicity. Collectively, these findings highlight *Glycosmis*-derived phytochemicals as promising natural scaffolds for the development of novel ALK-targeted anticancer agents. Overall, this study demonstrates that the integration of ML-driven virtual screening with advanced free energy calculations provides an efficient and reliable strategy for accelerating natural-product-based drug discovery. The identified compounds, particularly compound 54 and several bioactive alkaloids, are valuable candidates for future experimental validation, mechanistic investigations and structural optimization for the development of effective ALK inhibitors for cancer therapy. Further biochemical assays, kinase selectivity studies, cancer cell-based experiments, *in vivo* evaluations and structural optimization are required to confirm their inhibitory activity, mechanisms of action, safety and therapeutic relevance.

## Supplementary Material



## Data Availability

All data supporting the findings of this study are provided within the article and its supplementary material [75]. The input files and simulation parameters used for molecular dynamics (MD), steered molecular dynamics (SMD), and umbrella sampling (US) simulations have been deposited in Zenodo [76].
