# Supplementary material for: Discovery of potential ALK inhibitors from Glycosmis phytochemicals via integrated machine learning and umbrella sampling approaches
Source: R Soc Open Sci. 2026 Nov 4;13(11):261178. doi: 10.1098/rsos.261178 (PMC13730657; doi:10.1098/rsos.261178)
Supplement: Supplementary file 1 [file rsos.261178.zip › rsos.261178/rsos261178_si_001.docx]

Discovery of Potential ALK Inhibitors from *Glycosmis Phytochemicals* via Integrated Machine Learning and Umbrella Sampling Approaches

**Nguyen Xuan Ha^1^, Dau Xuan Duc^2^, Nguyen Thi Minh Hue^3^, Tran Ngoc Dung^3^, Nguyen Van Trang^4^, Doan Le Binh^2^, Le Thi My Chau^5^, and Phan Thi Thuy^2,*^**

*^1^Institute of Chemistry, Vietnam Academy of Science and Technology (VAST), Hanoi, Vietnam*

*^2^Department of Chemistry, Vinh University, 182 Le Duan, Truong Vinh, Nghean 43000, Vietnam*

*^3^Faculty of Chemistry and Center for Computational Science, Ha Noi National University of Education, 136 Xuan Thuy, Hanoi 10000, Vietnam*

*^4^Institute of Materials Science, VAST, 18 Hoang Quoc Viet, Hanoi 10000, Vietnam*

*^5^Institute of Biology, Chemistry and Environment, Vinh University, 182 Le Duan, Truong Vinh province, Nghean 43000, Vietnam*

Table S1. List of predicted binding affinities of EMNPD compounds with ALK using the CatBoost machine learning model

| **ID** | **SMILES** | **ΔG_ML_ (kcal/mol)** |
| --- | --- | --- |
| 1 | C1=CC2=C(C=C1)N(C1=C(C2=O)C(=CC=C1)O)C | -9.0991 |
| 2 | C1=CC2=C(C=C1)N(C1=C(C2=O)C(=CC(=C1OC)OC)O)C | -10.3787 |
| 3 | C1=CC2=C(C(=C1O)OC)N(C1=C(C2=O)C(=CC(=C1OC)OC)O)C | -10.7794 |
| 4 | C1=CC2=C(C(=C1)O)N(C1=C(C2=O)C(=C(C(=C1)O)OC)O)C | -10.4726 |
| 5 | C1=CC2=C(C(=C1O)OC)N(C1=C(C2=O)C(=C(C(=C1)O)OC)O)C | -10.8397 |
| 6 | C1=CC2=C(C=C1)N(C1=C(C2=O)C(=C(C(=C1)OC)OC)O)C | -10.4835 |
| 7 | C1=CC2=C(C(=C1)O)N(C1=C(C2=O)C(=C(C(=C1)OC)OC)O)C | -10.8697 |
| 8 | C1=CC2=C(C=C1)N(C1=C(C2=O)C(=C(C(=C1)OC)CC=C(C)C)O)C | -10.5504 |
| 9 | C1=CC2=C(C(=C1)O)N(C1=C(C2=O)C(=C(C(=C1)O)C/C=C(/CCC=C(C)C)\C)O)C | -10.0513 |
| 10 | C1(=C(C=C(C(=C1C)O)C(=O)/C=C/C1=CC=CN1)OC)OC | -11.0256 |
| 11 | C1(=C(C(=C2C(=C1)OC(=CC2=O)C1=CC(=C(C=C1)O)COC)O)[C@@H]1O[C@@H]([C@H]([C@@H]([C@H]1O)O)O)CO)O | -11.1342 |
| 12 | C1(=C(C(=C2C(=C1)OC(=CC2=O)C1=CC=C(C=C1)O)OC)[C@@H]1O[C@@H]([C@H]([C@@H]([C@H]1O)O)O)CO)O | -11.1295 |
| 13 | C1=CC2=C(C=C1)NC1=C3C(=CC(=C1C2=O)O)O[C@](C=C3)(C)CO | -10.9162 |
| 14 | C12=C(C(=O)C[C@H](O1)C1=CC=C(C=C1)OC)C(=CC1=C2C=CC(O1)(C)C)OC | -10.6933 |
| 15 | C1=CC2=C(C=C1)N(C1=C3C(=CC(=C1C2=O)O)OC(C=C3)(C)C)C | -10.6309 |
| 16 | C1(=C(C(=C2C(=C1[C@H]1[C@H]([C@@H]([C@@H]([C@H](O1)CO)O)O)O[C@@H]1[C@@H]([C@@H]([C@@H]([C@H](O1)CO)O)O)O)OC(=CC2=O)C1=CC(=C(C(=C1)OC)O)OC)O)[C@H]1[C@@H]([C@@H]([C@@H](CO1)O)O)O)O | -11.0365 |
| 17 | C1(=C(C(=CC(=C1)[C@@H](O)CCO)OC)O[C@H]1[C@@H]([C@H]([C@@H]([C@H](O1)CO)O)O)O)OC | -11.2437 |
| 18 | C1(=CC=C(C=C1)O)/C=C/C(=O)O | -9.61739 |
| 19 | C1=C2C(=C3C(=C1)C(=O)C1=C(N3C)C3=C(C=C1O)OC(C=C3)(C)C)OC[C@@](O2)(C)/C=C/C1=C(C=C(C2=C1N(C1=C(C2=O)C=CC=C1O)C)O)O | -9.82245 |
| 20 | C1=C2C(=C3C(=C1)C(=O)C1=C(N3C)C3=C(C=C1O)OC(C=C3)(C)C)OC[C@@](O2)(C)/C=C/C1=C(C=C(C2=C1N(C1=C(C2=O)C=CC=C1O)C)O)OC | -9.76581 |
| 21 | C1(=C(C2=C(C(=C1)O)C(=O)C[C@H](O2)C1=CC=C(C(=C1)O)OC)CC=C(C)C)OC | -11.1082 |
| 22 | C1=CC2=C(C(=C1O)OC[C@@]1(OC3=CC(=C4C(=C3C=C1)N(C1=C(C4=O)C=CC=C1O)C)O)C)N(C1=C3C(=CC(=C1C2=O)O)OC(C=C3)(C)C)C | -9.72641 |
| 23 | C1=CC2=C(C(=C1)O)N(C1=C(C2=O)C(=C2C(=C1CCC=C(C)C)OC(C=C2)(C)C)O)C | -10.7417 |
| 24 | C1=CC(=C2C(=C1)C(=O)C1=C(N2C)C2=C(C3=C1OC(C(=C3)O)(C)C)OC(C=C2)(C)C)O | -10.6297 |
| 25 | C1=CC(=C2C(=C1)C(=O)C1=C(N2C)C2=C(C3=C1OC=C3)OC(C=C2)(C)C)O | -10.7461 |
| 26 | C1(=C(C(=C2C(=C1)OC(=CC2=O)C1=CC=C(C=C1)O)OC)[C@@H]1O[C@@H]([C@H]([C@@H]([C@H]1O)O)O)CO)O | -11.1295 |
| 27 | C1=CC2=C(C=C1)N(C1=C(C2=O)C(=CC(=O)C1(CC=C(C)C)CC=C(C)C)O)C | -8.85722 |
| 28 | C1(=C(C2=C(C(=C1)O)C[C@@H]([C@H](O2)C1=CC(=C(C(=C1)O)OC)O)O)CNC(=O)SC)O | -11.2484 |
| 29 | C12=CC=C(C=C2NC2=CC=CC=C12)OC | -9.59878 |
| 30 | C1(=C(C2=C(C(=C1)OC)C(=O)C[C@H](O2)C1=CC=C(C(=C1)O)OC)CC=C(C)C)OC | -11.0182 |
| 31 | C12=CC(=CC=C2NC2=CC=CC=C12)C=O | -9.27134 |
| 32 | C1(=C(C=C(C(=C1C)O)C(=O)/C=C/C1=CC=CN1)OC)OC | -11.0256 |
| 33 | C12=CC(=CC=C2NC2=CC=CC(=C12)OC)C | -9.68791 |
| 34 | C12=CC(=C(C=C2NC2=CC=CC=C12)O)C | -9.62681 |
| 35 | C12=CC(=C(C=C2NC2=CC=C(C=C12)OC(=O)C)OC)C | -10.2376 |
| 36 | C12=CC(=CC=C2NC2=CC=CC=C12)C(=O)OC | -9.93828 |
| 37 | C12=CC(=C(C=C2NC2=CC=C(C(=C12)/C=C/C(=O)C)OC)O)C | -10.8665 |
| 38 | C12=C(C(=CC=C2NC2=CC=C(C=C12)C)O)CC=C(C)C | -9.84499 |
| 39 | C12=CC(=C(C=C2NC2=CC=CC(=C12)OC)OC)C | -10.0774 |
| 40 | C12=CC(=C(C=C2NC2=CC=C(C(=C12)CC=C(C)C)OC)O)C | -10.8015 |
| 41 | C12=CC(=C(C=C2NC2=CC=C(C(=C12)CC=C(C)C)O)O)C | -10.6451 |
| 42 | C12=CC(=C(C=C2NC2=CC=CC=C12)OC)C=O | -9.83461 |
| 43 | C12=CC(=CC=C2NC2=CC=C(C=C12)OC)C | -9.46914 |
| 44 | C1(=CC(=C2C(=C1)OC(=CC2=O)C1=CC=C(C(=C1)O)OC)O[C@@H]1O[C@@H]([C@H]([C@@H]([C@H]1O)O)O)CO)O | -11.1488 |
| 45 | C12=CC(=C(C=C2NC2=CC=C(C=C12)OC)OC)C=O | -10.2468 |
| 46 | C12=CC(=C(C=C2NC2=CC=C(C=C12)OC)OC)C | -10.1354 |
| 47 | C12=CC(=CC=C2NC2=CC=CC(=C12)OC)C | -9.68791 |
| 48 | C1=CC2=C(C=C1)NC(=O)C1=C2C=C(C=C1)C | -9.17936 |
| 49 | C12=CC(=C(C=C2NC2=CC=CC(=C12)OC)O)C | -10.4667 |
| 50 | C1(=CC(=CC(=C1O[C@@H]1O[C@@H]([C@H]([C@@H]([C@H]1O)O)O)CO)OC)/C=C/CO)[C@@H](CO)CC1=CC(=C(C=C1)O)OC | -11.0838 |
| 51 | C12=CC(=C(C=C2NC2=CC=C(C=C12)OC)O)C | -10.5139 |
| 52 | C12=CC(=C(C=C2NC2=CC=CC=C12)OC)C | -9.56563 |
| 53 | C1(=CC2=C(C(=C1)O)C(=O)C[C@@H](O2)C1=CC(=C(C=C1)O)OC)O[C@@H]1O[C@@H]([C@H]([C@@H]([C@H]1O)O)O)CO | -11.3471 |
| 54 | C1(=CC2=C(C(=C1)O)C(=O)C[C@@H](O2)C1=CC(=C(C=C1)O)OC)O[C@@H]1O[C@@H]([C@H]([C@@H]([C@H]1O)O)O)CO[C@H]1O[C@@H]([C@H]([C@@H]([C@H]1O)O)O)C | -11.1081 |
| 55 | C12=C(NC3=C1C=CC=C3OC)C1=C(C(=C2)C)OC(CC1)(C)C | -10.8175 |
| 56 | C12=C(NC3=C1C=CC=C3O)C1=C(C(=C2)C=O)OC(CC1)(C)C | -10.9875 |
| 57 | C12=C(NC3=C1C=CC=C3OC)C1=C(C(=C2)C=O)OC(CC1)(C)C | -10.8026 |
| 58 | C1(=CC2=C(C(=C1CNC(=O)SC)O)C[C@@H]([C@H](O2)C1=CC(=C(C(=C1)O)OC)O)O)O | -11.2614 |
| 59 | C12=C(NC3=C1C=C(C(=C3)O)C)C=CC1=C2C=CC(O1)(C)C | -10.9735 |
| 60 | C1(=CC=C(C(=C1)OC)O)C(=O)OC[C@@]1([C@H]([C@@H](OC1)O[C@@H]1[C@H]([C@@H]([C@H](O[C@H]1OC1=C(C=C(C=C1OC)O)OC)CO)O)O)O)O | -11.0863 |
| 61 | C1(=CC=C(C(=C1)OC)O)C(=O)OC[C@@]1([C@H]([C@@H](OC1)O[C@@H]1[C@H]([C@@H]([C@H](O[C@H]1OC1=CC(=C(C=C1)O)OC)CO)O)O)O)O | -11.2323 |
| 62 | C12=CC(=C(C(=C2NC2=CC=CC(=C12)OC)[C@@H]1[C@H]([C@@](CO1)(C)O)C1=CNC2=C1C=CC=C2CC=C(C)C)O)C | -9.28604 |
| 63 | C12=CC(=C(C(=C2NC2=CC=C(C(=C12)CC=C(C)C)OC)C1=C2C(=CC(=C1O)C)C1=C(C(=CC=C1N2)OC)CC=C(C)C)O)C | -8.58484 |
| 64 | C12=CC(=C(C(=C2NC2=CC=CC(=C12)OC)C1=C2C(=CC(=C1O)C)C1=C(C=CC=C1N2)OC)O)C | -9.24639 |
| 65 | C12=CC(=CC(=C2NC2=CC=CC=C12)OC)C=O | -9.81179 |
| 66 | C12=CC=C3C(=C2NC2=C1C=C(C(=C2)O)C)C=C[C@@](O3)(CCC=C(C)C)C | -10.8564 |
| 67 | C12=CC=C3C(=C2NC2=C1C=C(C(=C2C1=C(C(=CC2=C1NC1=C4C(=CC=C21)O[C@](C=C4)(C)CCC=C(C)C)C)O)O)C)C=C[C@@](O3)(CCC=C(C)C)C | -8.66654 |
| 68 | C12=C(NC3=CC=CC(=C13)OC)C(=O)C[C@H](C2)C | -10.4448 |
| 69 | C1=CC=C2C(=C1)C(=C1C(=N2)OC=C1)OC | -9.26911 |
| 70 | C1=C(C(=C2C(=C1)C(=C1C(=N2)OC=C1)OC)OC)OC | -10.1326 |
| 71 | C1(=C(C=C2C(=C1)C(=C1C(=N2)OC=C1)OC)OC)OC | -9.84592 |
| 72 | C1=CC(=C2C(=C1)C(=C1C(=N2)OC=C1)OC)OC | -9.64899 |
| 73 | C1=CC(=C2C(=C1)C(=C1C(=N2)OC=C1)OC)O | -10.1969 |
| 74 | C1=C(C(=C2C(=C1)C(=C1C(=N2)OC=C1)OC)OC)O | -10.4551 |
| 75 | C1=CC=C2C(=C1)C(=C1C(=N2)OC(=C1)C(C)(C)O)OC | -9.97603 |
| 76 | C1=CC2=C(C=C1)N(C1=C(C2=O)C=CO1)C | -9.38849 |
| 77 | C1=CC2=C(C(=C1)OC)N(C1=C(C2=O)C=CO1)C | -9.90633 |
| 78 | C1=C(C2=C(C=C1OC)N(C1=C(C2=O)C=CO1)C)OC | -10.5548 |
| 79 | C1=C(C(C2=C(C1=O)[C@H](C1=C(N2)OC=C1)OC)(OC)OC)OC | -9.55004 |
| 80 | C1=CC=C(C2=C1C(=C(C(=O)N2C)CC=C(C)C)OC)OC | -10.5281 |
| 81 | C12=C(C3=C(C(=C1)O)C(=O)[C@@H]([C@H](O3)C1=CC=C(C(=C1)O)OC)O)C=CC(O2)(C)C | -11.1473 |
| 82 | C1=CC=C(C2=C1C(=C(C(=O)N2)CC=C(C)C)OC)OC | -10.0862 |
| 83 | C1=CC=CC2=C1C(=C(C(=O)N2C)/C=C/C(C)(O)C)OC | -10.3043 |
| 84 | C1=CC=C(C2=C1C(=C(C(=O)N2C)/C=C/C(C)(O)C)OC)O | -10.7556 |
| 85 | C1=CC=C(C2=C1C(=C(C(=O)N2C)/C=C/C(C)(O)C)OC)OC | -10.7586 |
| 86 | C1=CC=C(C2=C1C(=C(C(=O)N2C)C[C@@H](C(=C)C)OO)OC)OC | -10.9027 |
| 87 | C1[C@@H](CC2=CC[C@@H]3[C@@H]([C@]2(C1)C)CC[C@]1([C@@H]3CC[C@@H]1[C@@H](CC[C@H](C(C)C)CC)C)C)O | -9.7764 |
| 88 | C1=CC=CC2=C1C(=C(C(=O)N2)/C=C/C(=C)C)OC | -9.87752 |
| 89 | C1=CC2=C(C=C1)N(C(=O)C=C2OC/C=C(/CCC=C(C)C)\C)C | -9.36605 |
| 90 | C1=CC2=C(C=C1)N(C(=O)C=C2OC/C=C(/C/C=C/C(C)(C)OO)\C)C | -9.2846 |
| 91 | C1=CC2=C(C=C1)N(C(=O)C=C2OC/C=C(/CC[C@@H](C(=C)C)O)\C)C | -9.53817 |
| 92 | C1=CC2=C(C=C1)N(C(=O)C=C2OC/C=C(/CC[C@@H](C(=C)C)OO)\C)C | -9.50282 |
| 93 | C1=CC2=C(C(=C1OC)OC)N(C(=O)C1=C2OC(C=C1)(C)C)C | -10.9306 |
| 94 | C1=CC2=C(C=C1)N(C(=O)C=C2OC)C | -9.69776 |
| 95 | C12=CC(=C(C(=C2NC2=C(C=CC=C12)O)CC=C(C)C)O)C=O | -11.0243 |
| 96 | C1(=CC2=C(C=C1)N(C(=O)C=C2OC)C)O | -10.0762 |
| 97 | C1=CC2=C(C=C1)N(C=NC2=O)C | -10.0517 |
| 98 | C1=CC2=C(C=C1)N(C(=NC2=O)CC1=CC=CC=C1)C | -8.85727 |
| 99 | C1=CC2=C(C=C1)C(=O)N=C(N2)CC1=CC=CC=C1 | -9.14871 |
| 100 | C1=CC2=C(N=C(N=C2C=C1)CC1=CC=CC=C1)OC | -8.9142 |
| 101 | C1=CC=C2C(=C1)C(=O)N(C=N2)/C=C\C1=CC=CC=C1 | -9.17951 |
| 102 | C1=CC2=C(C=C1)N(C(=O)NC2=O)C | -10.1136 |
| 103 | C1=CC2=C(C=C1)N([C@H](NC2=O)CC1=CC=CC=C1)C | -9.2374 |
| 104 | C1=CC2=C(C=C1)N([C@H](NC2=O)CC1=CC=C(C=C1)OC)C | -9.3031 |
| 105 | C1=CC=C2C(=C1)C(=O)NC(=N2)CC1=CC=CC=C1 | -9.11218 |
| 106 | C1(=CNC2=C1C=CC=C2)CCN(C(=O)SC)C | -9.39487 |
| 107 | C1(=CNC2=C1C=CC=C2)CCNC(=O)SC | -9.07649 |
| 108 | C1(=CNC2=C1C=CC=C2CC=C(C)C)C/C=C(/CO)\C | -10.5965 |
| 109 | C1(=CNC2=C1C=CC=C2CC=C(C)C)CC=C(CO)CO | -10.7798 |
| 110 | C1(=CNC2=C1C=CC=C2CC=C(C)C)[C@H]1COC[C@@]1(C)O | -10.73 |
| 111 | C1(=CNC2=C1C=CC=C2CC=C(C)C)CC=C(C)C | -9.43961 |
| 112 | C1(=O)C=C(C2=C([C@@]1(O)CC(=O)C)C(=C1C(=N2)OC=C1)OC)OC | -10.574 |
| 113 | C1=C([C@](C2=NC3=C(C(=C2C1=O)OC)C=CO3)(CC(=O)C)O)OC | -10.6557 |
| 114 | C12=C(C(=C3C(=N1)OC=C3)OC)[C@@H](OC2=O)CC(=O)OC | -9.93567 |
| 115 | C1(=CC(=C2C(=C1)OC(=CC2=O)C1=CC=CC=C1)O)O | -9.49867 |
| 116 | C1(=CC(=C2C(=C1)OC(=C(C2=O)O)C1=CC=C(C(=C1)O)O)O)O | -10.2292 |
| 117 | C1(=CC(=C2C(=C1)OC(=C(C2=O)O)C1=CC=C(C=C1)O)O)O | -10.169 |
| 118 | C1(=CC(=C2C(=C1[C@@H]1O[C@@H]([C@H]([C@@H]([C@H]1O)O)O)CO)OC(=CC2=O)C1=CC=C(C=C1)O)O)O | -10.9198 |
| 119 | C12=CC(=C(C=C2NC2=CC=C(C=C12)O)OC)C | -10.5536 |
| 120 | C1=CC2=C(C(=C1)O)N(C1=C(C2=O)C(=C(C(=C1CC=C(C)C)O)CC=C(C)C)O)C | -9.78359 |
| 121 | C1(=CC(=C2C(=C1[C@@H]1O[C@@H]([C@H]([C@@H]([C@H]1O)O)O)CO)OC(=CC2=O)C1=CC(=C(C=C1)O)O)O)O | -10.6826 |
| 122 | C12=CC3=C(C(=C1O[C@@H]([C@H]2O)C(C)(C)O)OC)N(C1=C(C3=O)C(=CC(=C1OC)OC)O)C | -11.0627 |
| 123 | C1(=CC(=C2[C@H](C1)OC(=CC2=O)C1=CC(=C(C=C1)O[C@@H]1O[C@@H]([C@@H]([C@@H]([C@H]1O[C@@H]1O[C@H]([C@@H]([C@H]([C@H]1O)O)O)C)O)O)CO[C@@H]1O[C@H]([C@@H]([C@H]([C@H]1O)O)O)C)O)O)O | -10.8197 |
| 124 | C1(=CC2=C(C(=C1)O)C(=O)C[C@@H](O2)C1=CC(=C(C=C1)O)OC)O | -10.6618 |
| 125 | C12=CC(=C3C(=C2NC2=C1C=CC=C2)C=CC(O3)(C)C)C | -10.402 |
| 126 | C12=CC(=C3C(=C2NC2=C1C=CC=C2OC)C=CC(O3)(C)C)C | -10.9871 |
| 127 | C1(=C2C(=C3C(=C1)O[C@H]([C@@H](C3)O)C1=CC(=C(C(=C1)OC)OC)OC)OC(=O)C=C2)O | -10.7911 |
| 128 | C1(=C2C(=C3C(=C1)O[C@H]([C@@H](C3)O)C1=CC(=C(C(=C1)O)OC)O)OC(=O)C=C2)O | -10.8189 |
| 129 | C1=C(C(=C(C=C1CCO)O)OC)O[C@H]1[C@@H]([C@H]([C@@H]([C@H](O1)CO)O)O)O | -11.0471 |
| 130 | C12=C(C3=C(C(=C1)O)C[C@H]([C@@H](O3)C1=CC(=C(C(=C1)O)OC)O)O)C=CC(=O)O2 | -10.8631 |
| 131 | C1(=C(C2=C(C(=C1)O)C(=O)C[C@H](O2)C1=CC=C(C=C1)O)CC=C(C)C)O | -10.5809 |
| 132 | C1(=C(C2=C(C(=C1)O)C(=O)C[C@@H](O2)C1=CC=C(C=C1)OC)CC=C(C)C)O | -10.6862 |
| 133 | C1(=C(C2=C(C(=C1)O)C(=O)C[C@@H](O2)C1=CC=C(C=C1)O)CC=C(C)C)OC | -10.5501 |
| 134 | C1=C(C=C(C(=C1OC)O[C@H]1[C@@H]([C@H]([C@@H]([C@H](O1)CO)O)O)O)OC)O[C@H]1[C@@H]([C@H]([C@@H]([C@H](O1)COC(=O)C1=CC(=C(C(=C1)OC)O)OC)O)O)O | -11.139 |
| 135 | C1=C(C=C(C(=C1OC)O[C@H]1[C@@H]([C@H]([C@@H]([C@H](O1)CO)O)O)O)OC)O[C@H]1[C@@H]([C@H]([C@@H]([C@H](O1)COC(=O)C1=CC=C(C(=C1)OC)O)O)O)O | -11.0312 |
| 136 | C1(=C(C(=C2C(=C1)OC=C(C2=O)C1=CC(=C(C=C1)OC)O)OC)OC)O[C@H]1[C@@H]([C@H]([C@@H]([C@H](O1)CO[C@H]1[C@@H]([C@@](CO1)(O)COC(=O)/C=C/C1=CC=C(C=C1)O)O)O)O)O | -9.92937 |
| 137 | C1(=C(C(=C2C(=C1)OC=C(C2=O)C1=C(C=C(C(=C1)OC)OC)O)OC)OC)O[C@H]1[C@@H]([C@H]([C@@H]([C@H](O1)CO[C@H]1[C@@H]([C@@](CO1)(O)COC(=O)/C=C/C1=CC=C(C=C1)O)O)O)O)O | -9.65785 |
| 138 | C1(=C(C(=C2C(=C1)OC=C(C2=O)C1=C(C=C(C(=C1)OC)OC)O)OC)OC)O[C@H]1[C@@H]([C@H]([C@@H]([C@H](O1)CO[C@H]1[C@@H]([C@@](CO1)(O)CO)O)O)O)O | -10.9494 |
| 139 | C1(=CC=C2C(=C1OC)OC=C(C2=O)C1=CC=C(C=C1)OC)O[C@H]1[C@@H]([C@H]([C@@H]([C@H](O1)CO[C@H]1[C@@H]([C@@](CO1)(O)CO)O)O)O)O | -10.137 |
| 140 | C1(=C(C=C2C(=C1)OC=C(C2=O)C1=CC=C(C=C1)OC)OC)O[C@H]1[C@@H]([C@H]([C@@H]([C@H](O1)CO[C@]1([C@@](COC1)(O)CO)O)O)O)O | -10.0692 |
| 141 | C1(=CC2=C(C(=C1)O)C(=O)C[C@H](O2)C1=CC=C(C=C1)OCC=C(C)C)O | -10.2866 |
| 142 | C1(=CC2=C(C(=C1)O)C(=O)C[C@H](O2)C1=CC=C(C=C1)OCC=C(C)C)OC | -10.3707 |
| 143 | C1(=C(C2=C(C(=C1)O)C[C@@H]([C@H](O2)C1=CC(=C(C(=C1)O)OC)O)O)CC1=C(C2=C(C=C1O)O[C@@H]([C@H](C2)O)C1=CC(=C(C(=C1)O)OC)O)O)O | -9.48356 |
| 144 | C1(=C(C2=C(C(=C1)OC)C(=O)C[C@H](O2)C1=CC=C(C=C1)OC)/C=C/C(C)(C)O)OC | -10.853 |
| 145 | C1=CC(=C(C2=C1C(=C(C(=O)N2)C=O)OC)OC)OC | -10.8505 |
| 146 | C1(=CC2=C(C(=C1C/C=C(/CCC=C(C)C)\C)O)C(=O)C[C@H](O2)C1=CC=C(C=C1)OC)O | -9.58943 |
| 147 | C1=CC2=C(C(=C1O)OC)N(C1=C3C(=CC(=C1C2=O)O)OC(C=C3)(C)C)C | -11.1873 |
| 148 | C1(=C(C2=C(C(=C1)O)C[C@@H]([C@H](O2)C1=CC(=C(C(=C1)O)OC)O)O)CC1=C(C=C(C2=C1O[C@@H]([C@H](C2)O)C1=CC(=C(C(=C1)O)OC)O)O)O)O | -9.36281 |
| 149 | C1(=C(C2=C(C(=C1)O)C(=O)[C@@H]([C@H](O2)C1=CC=C(C=C1)O)O)CC=C(C)C)OC | -10.7807 |
| 150 | C1(=C(C2=C(C(=C1)O)C(=O)[C@@H]([C@H](O2)C1=CC=C(C=C1)O)O)C/C=C(/CCC=C(C)C)\C)O | -9.38912 |
| 151 | C1(=C(C2=C(C(=C1)O)C(=O)C[C@H](O2)C1=CC=C(C=C1)OC)C/C=C(/CCC=C(C)C)\C)O | -9.52562 |
| 152 | C1=CC2=C(C3=C1O[C@](CO3)(C)/C=C/C1=C(C=C(C3=C1N(C1=C(C3=O)C=CC=C1O)C)O)O)N(C1=C(C2=O)C(=CC(=C1OC)OC)O)C | -9.83533 |
| 153 | C12=CC(=CC=C2NC2=CC=CC=C12)C | -8.91517 |
| 154 | C12=CC=CC=C2NC2=CC=CC=C12 | -8.76831 |
| 155 | C12=C(NC3=C1C=C(C=C3)C)C=CC1=C2C=CC(O1)(C)C | -10.298 |
| 156 | C12=CC(=C(C(=C1C=CC(O2)(C)C)O)C(=O)/C=C/C1=CC=C(C=C1)OC)OC | -10.7308 |
| 157 | C12=CC(=C(C(=C1C=CC(O2)(C)C)O)C(=O)/C=C/C1=CC=C(C(=C1)OC)OC)OC | -10.9358 |
| 158 | C1=CC2=C(C(=C1)O)N(C1=C(C2=O)C(=C(C(=C1OC)O)CC=C(C)C)O)C | -11.088 |
| 159 | C12=CC(=CC(=C1C(=O)C(=CO2)C1=CC(=C(C=C1)O[C@H]1[C@@H]([C@H]([C@@H]([C@H](O1)CO[C@H]1[C@@H]([C@](CO1)(CO)O)O)O)O)O)OC)O)OC | -10.7684 |
| 160 | C1=CC2=C(C(=C1)O)NC1=C3C(=CC(=C1C2=O)O)OC(C=C3)(C)C | -11.2227 |
| 161 | C12=C(C(=O)C[C@H](O1)C1=CC=C(C(=C1)OC)OC)C(=CC1=C2C=CC(O1)(C)C)OC | -10.79 |
| 162 | C12=C(C[C@@H]([C@H](O1)C1=CC(=C(C(=C1)O)OC)O)O)C(=CC(=C2)O)O | -10.5719 |
| 163 | C1=CC(=C(C=C1)O)C(=O)O | -9.58407 |
| 164 | C1=CC2=C(C(=C1O)OC)N(C1=C3C(=CC(=C1C2=O)O)OC(C=C3)(C)C)C | -11.1873 |
| 165 | C1=CC2=C(C(=C1O)OC)NC1=C3C(=CC(=C1C2=O)O)OC(C=C3)(C)C | -11.4927 |
| 166 | C1(=CC(=C(C=C1)O)O)C(=O)O | -10.1269 |
| 167 | C1(=CC=C(C=C1)O)C(=O)O | -9.99271 |
| 168 | C1(=CC(=C(C=C1)O)OC)C(=O)O | -10.2244 |
| 169 | C1=C(C(=C(C=C1CCO)O)OC)O | -10.0176 |
| 170 | C1=C(C(=C(C=C1/C=C/CO)O)OC)OC | -10.337 |
| 171 | C12=C(C3=C(C(=C1)O)C[C@H]([C@@H](O3)C1=CC(=C(C(=C1)OC)OC)OC)O)C=CC(=O)O2 | -10.8756 |
| 172 | C1(=CC=C(C=C1)O)O[C@H]1[C@@H]([C@H]([C@@H]([C@H](O1)CO)O)O)O | -10.0817 |
| 173 | C1(=C(C=C(C=C1OC)O)OC)O[C@H]1[C@@H]([C@H]([C@@H]([C@H](O1)CO)O)O)O | -10.9722 |
| 174 | C1(=CC(=C(C=C1)O)OC)O[C@H]1O[C@H]([C@@H]([C@H]([C@@H]1O[C@H]1[C@@H]([C@@](CO1)(O)CO)O)O)O)CO | -10.6562 |
| 175 | C1(=CC(=C(C=C1)O)OC)O[C@H]1[C@@H]([C@H]([C@@H]([C@H](O1)CO)O)O)O | -10.4283 |
| 176 | [C@H]1([C@H]([C@@H]([C@H]([C@@H](O1)OC1=CC=C(C=C1OC)CCO)O)O)O)CO | -10.7125 |
| 177 | O1[C@@H]([C@H]([C@H]([C@@H]([C@@H]1CO)O)O)O[C@H]1[C@H]([C@@](CO1)(O)COC(=O)C1=CC(=C(C=C1)O)OC)O)OC1=CC=C(C(=C1)OC)O | -10.7596 |
| 178 | C1(=C(C2=C(C(=C1)OC)C(=O)C[C@@H](O2)C1=CC=C(C=C1)OC)CC=C(C)C)OC | -10.4749 |
| 179 | C1(=C(C2=C(C(=C1)O)C(=O)C[C@@H](O2)C1=CC=C(C=C1)OC)CC=C(C)C)OC | -10.7019 |
| 180 | C1=CC2=C(C=C1)NC1=C3C(=CC(=C1C2=O)O)O[C@H](C=C3)C | -10.8183 |
| 181 | [C@H]1([C@H]([C@@H]([C@H]([C@@H](O1)OC1=C(C=C(C=C1)[C@H](O)[C@H](CO)O)OC)O)O)O)COC(=O)C1=CC=C(C(=C1)OC)O | -10.7312 |
| 182 | [C@H]1([C@H]([C@@H]([C@H]([C@@H](O1)OC1=C(C=C(C=C1OC)[C@@H](O)[C@H](O)CO)OC)O)O)O)CO | -10.9792 |
| 183 | C([C@H]([C@H](C1=CC(=C(C=C1)O)OC)O[C@@H]1O[C@@H]([C@H]([C@@H]([C@H]1O)O)O)CO)OC1=C(C=C(C=C1OC)[C@H]1[C@@H]([C@@H](CO1)CC1=CC=C(C(=C1)OC)O)CO)OC)O | -10.2954 |
| 184 | C12=CC(=C(C=C2NC2=C(C=CC=C12)OC)O)C | -10.3984 |
| 185 | C1(=CC(=CC(=C1O[C@@H]1O[C@@H]([C@H]([C@@H]([C@H]1O)O)O)CO)OC)/C=C/CO)OC | -10.66 |
| 186 | C1=CC(=C(C=C1O)O)/C=C/C1=CC(=CC(=C1)O)O | -9.76178 |
| 187 | [C@H]12[C@@H](CC1(C)C)C(=C)CC/C=C(/CC2)\C | -9.36919 |
| 188 | C1(=CC(=O)[C@H]2C([C@@H]1C2)(C)C)C | -9.09147 |
| 189 | CC(CCC[C@H](CCC[C@H](CCC/C(=C/CO)/C)C)C)C | -9.79251 |
| 190 | OC/C=C(\C)/CCC[C@@H](CCC[C@@H](CCCC(C)C)C)C | -9.67918 |
| 191 | C1C(=O)C=C2[C@](C1)([C@@H]1[C@@H](CC2=O)[C@@H]2[C@](CC1)([C@H](CC2)[C@@H](CC[C@H](C(C)C)C)C)C)C | -10.1974 |
| 192 | C1C(=O)C=C2[C@](C1)([C@@H]1[C@@H](CC2=O)[C@@H]2[C@](CC1)([C@H](CC2)[C@@H](CC[C@H](C(C)C)CC)C)C)C | -10.1873 |
| 193 | C1C(=O)C=C2[C@](C1)([C@@H]1[C@@H](CC2)[C@@H]2[C@](CC1)([C@H](CC2)[C@@H](CC[C@H](C(C)C)CC)C)C)C | -9.64661 |
| 194 | C1[C@@H](CC2=CC[C@@H]3[C@@H]([C@]2(C1)C)CC[C@]1([C@H]3CC[C@@H]1[C@@H](/C=C/[C@H](C(C)C)CC)C)C)O | -10.0718 |
| 195 | C1[C@@H](C[C@H]2[C@](C1)([C@@H]1C(=CC2)[C@H]2[C@](CC1)([C@H](CC2)[C@@H](/C=C/[C@H](C(C)C)CC)C)C)C)O | -9.97148 |
| 196 | C1=CC=C(C2=C1C(=C(C(=O)N2C)C[C@@H](C(=C)C)O)OC)OC | -11.0416 |
| 197 | C1[C@@H](C[C@@H]2[C@]3(C1)[C@]1([C@@H](CC2)[C@]2([C@](CC1)([C@H](CC2)[C@@H](CC[C@H](C(C)C)C)C)C)C)C3)O | -9.95775 |
| 198 | C1[C@H](C([C@H]2[C@](C1)([C@@H]1[C@@](CC2)([C@]2(C(=CC1)[C@H]1[C@@]([C@H](C2)O)(CCC(C1)(C)C)C)C)C)C)(C)C)O | -10.5471 |
| 199 | C1[C@H](C([C@H]2[C@](C1)(C1=CC[C@]3([C@]([C@@H]1CC2)(CC[C@@H]3[C@@H](CC[C@@H](C(=C)C)C)C)C)C)C)(C)C)O | -9.73113 |
| 200 | C1[C@@H](C([C@H]2[C@](C1)(C1=CC[C@]3([C@]([C@@H]1CC2)(CC[C@@H]3[C@@H](CC[C@@H](C(=C)C)C)C)C)C)C)(C)C)O | -9.73463 |
| 201 | C1[C@H](C([C@H]2[C@](C1)(C1=CC[C@]3([C@]([C@@H]1CC2)(CC[C@@H]3[C@@H](CCC(C(=C)C)(C)C)C)C)C)C)(C)C)O | -10.0756 |
| 202 | C1[C@@H](C([C@H]2[C@](C1)(C1=CC[C@]3([C@]([C@@H]1CC2)(CC[C@@H]3[C@@H](CCC(C(=C)C)(C)C)C)C)C)C)(C)C)O | -10.0908 |
| 203 | C1[C@H](C([C@H]2[C@](C1)(C1=CC[C@]3([C@]([C@@H]1CC2)(CC[C@@H]3[C@@H](CCC(C(=C)C)(C)C)C)C)C)C)(C)C)OC(=O)C | -10.249 |
| 204 | C1[C@@H](C([C@H]2[C@](C1)(C1=CC[C@]3([C@]([C@@H]1CC2)(CC[C@@H]3[C@@H](CCC(C(=C)C)(C)C)C)C)C)C)(C)C)OC(=O)C | -10.2732 |
| 205 | C1[C@H](C(C2=CC[C@@H]3[C@@]([C@H]2C1)(CC[C@]1([C@]3(CC[C@@]2([C@@H]1CC(CC2)(C)C)C)C)C)C)(C)C)O | -10.1833 |
| 206 | C1[C@@H](C([C@H]2[C@](C1)([C@@H]1[C@@](CC2)([C@]2(C(=CC1)[C@H]1[C@@](CC2)(CCC(C1)(C)C)C)C)C)C)(C)C)O | -10.1411 |
| 207 | C1[C@@H](C([C@H]2[C@](C1)([C@@H]1[C@@](CC2)([C@]2([C@H](CC1)[C@@H]1[C@@](CC2)(CC[C@H]1C(=C)C)C)C)C)C)(C)C)O | -10.1198 |
| 208 | C1[C@H](C([C@H]2[C@](C1)([C@@H]1[C@@](CC2)([C@]2(C(=CC1)[C@@H]1[C@@](CC2)(CCC(C1)(C)C)C(=O)O)C)C)C)(C)C)O | -10.975 |
| 209 | C1[C@H]2[C@@]([C@]34[C@@](C1)([C@@H](OC(=O)[C@H]3O4)C1=COC=C1)C)(C)C(=O)C[C@@H]1[C@@]32[C@@H](OC1(C)C)CC(=O)OC3 | -9.93803 |
| 210 | C1[C@H]2[C@@]([C@]34[C@@](C1)([C@@H](OC(=O)[C@H]3O4)C1=C[C@H](OC1=O)O)C)(C)C(=O)C[C@@H]1[C@@]32[C@@H](OC1(C)C)CC(=O)OC3 | -10.4292 |
| 211 | C1[C@H]2[C@@]([C@]34[C@@](C1)([C@@H](OC(=O)[C@H]3O4)C1=CC(=O)O[C@H]1O)C)(C)C(=O)C[C@@H]1[C@@]32[C@@H](OC1(C)C)CC(=O)OC3 | -10.4175 |
| 212 | CS/C=C/C(=O)NCCC1=CC=CC=C1 | -8.67985 |
| 213 | CS/C=C/C(=O)N(CCC1=CC=CC=C1)C | -8.3177 |
| 214 | CS/C=C\C(=O)N(C(=O)CC1=CC=CC=C1)C | -9.08536 |
| 215 | CS/C=C\C(=O)N(C(=O)CC1=CC=C(C=C1)OC)C | -9.46216 |
| 216 | CS/C=C/C(=O)N/C=C\C1=CC=CC=C1 | -8.59488 |
| 217 | CS/C=C/C(=O)N(/C=C\C1=CC=CC=C1)C | -8.7346 |
| 218 | CS/C=C/C(=O)N(C(=O)CC1=CC=CC=C1)C | -9.12634 |
| 219 | C1=CC=CC=C1CCN(C/C=C/SC)C=O | -8.41535 |
| 220 | CS/C=C\CN(CCC1=CC=CC=C1)C=O | -8.27758 |
| 221 | C[S@@](=O)/C=C/C(=O)NC | -8.94466 |
| 222 | C[S@@](=O)/C=C\C(=O)NC | -8.93851 |
| 223 | CS/C=C/C(=O)NC | -8.62927 |
| 224 | CS/C=C\C(=O)NC | -8.62017 |
| 225 | CS/C=C/C(=O)N/C=C/C1=CC=CC=C1 | -8.62566 |
| 226 | CS/C=C/C(=O)N(/C=C/C1=CC=CC=C1)C | -8.76593 |
| 227 | CS(=O)(=O)/C=C/C(=O)NCCC1=CC=C(C=C1)OCC=C(C)C | -10.3668 |
| 228 | CS(=O)(=O)/C=C/C(=O)N(CCC1=CC=C(C=C1)OCC=C(C)C)C | -10.13 |
| 229 | C1=CC(=CC=C1CCNC(=O)/C=C/S(=O)(=O)C)OCC=C(C)C | -10.3828 |
| 230 | C1=CC(=CC=C1CCNC(=O)/C=C/S(=O)(=O)C)OCC=C(C)C | -10.3828 |
| 231 | C1=CC(=CC=C1CCN(C(=O)/C=C/S(=O)(=O)C)C)OCC=C(C)C | -10.1433 |
| 232 | C1=CC(=CC=C1CCN(C(=O)/C=C/S(=O)(=O)C)C)OCC=C(C)C | -10.1433 |
| 233 | C1=CC(=C(C=C1CCNC(=O)/C=C/S(=O)(=O)C)O)OCC=C(C)C | -10.8009 |
| 234 | C1=CC(=C(C=C1CCNC(=O)/C=C/S(=O)(=O)C)OC)OCC=C(C)C | -10.81 |
| 235 | C1=CC(=C(C=C1CCNC(=O)/C=C/S(=O)(=O)C)OC)OC/C=C(/CC/C=C(\C)/CO)\C | -10.9085 |
| 236 | CSC(=O)N(C)CCC1=CC=CC=C1 | -8.59638 |
| 237 | CSC(=O)N(C)/C=C/C1=CC=CC=C1 | -8.99588 |
| 238 | CSC(=O)N(C)/C=C\C1=CC=CC=C1 | -8.99993 |
| 239 | CSC(=O)N(C)C(=O)CC1=CC=CC=C1 | -8.9403 |
| 240 | C(=C\C(=O)NCCC1=CC=C(C=C1)OC/C=C(/C1=CC(=O)C(O1)(C)C)\C)/S(=O)(=O)C | -9.59529 |
| 241 | C(=C\C(=O)NCCC1=CC=C(C=C1)OCC[C@H](C1=CC(=O)C(O1)(C)C)C)/S(=O)(=O)C | -10.2944 |
| 242 | C(=C\C(=O)NCCC1=CC=C(C=C1)OCC[C@H](C1=CC(=O)C(O1)(C)C)C)/[S@](=O)C | -10.1774 |
| 243 | C(CC(=O)NCCC1=CC=C(C=C1)OCC[C@H](C1=CC(=O)C(O1)(C)C)C)[S@](=O)C | -10.3733 |
| 244 | C(=O)(CCS(=O)(=O)C)N(C)CCC1=CC=C(C=C1)O | -10.6342 |
| 245 | C(=C\C(=O)NCCC1=CC=C(C=C1)OCC[C@H](C1=CC(=O)C(O1)(C)C)C)/[S@](=O)C | -10.1774 |
| 246 | CS(=O)(=O)/C=C/C(=O)NCCC1=CC=C(C=C1)OC/C=C(/CCC=C(C)C)\C | -9.91083 |
| 247 | CS(=O)(=O)/C=C/C(=O)N(CCC1=CC=C(C=C1)OC/C=C(/CCC=C(C)C)\C)C | -9.6163 |
| 248 | CS(=O)(=O)/C=C/C(=O)NCCC1=CC=C(C=C1)OC/C=C(/CC/C=C(\C)/CO)\C | -10.4424 |
| 249 | CS(=O)(=O)/C=C/C(=O)NCCC1=CC=C(C=C1)OC/C=C(/CC[C@@H](C(C)(C)O)O)\C | -10.886 |
| 250 | CS(=O)(=O)/C=C/C(=O)N(CCC1=CC=C(C=C1)OC/C=C(/CC/C=C(\C)/C=O)\C)C | -9.54905 |
| 251 | CS(=O)(=O)/C=C/C(=O)N(CCC1=CC=C(C=C1)OC/C=C(/CC(=O)C=C(C)C)\C)C | -9.52484 |
| 252 | CS(=O)(=O)/C=C/C(=O)N(CCC1=CC=C(C=C1)OCC/C(=C/C(=O)C=C(C)C)/C)C | -9.75934 |
| 253 | CS(=O)(=O)/C=C/C(=O)NCCC1=CC=C(C=C1)OC/C=C(/C1=CC(=O)C(O1)(C)C)\CO | -9.58934 |
| 254 | CS(=O)(=O)/C=C/C(=O)NCCC1=CC=C(C=C1)OC/C=C(\C1=CC(=O)C(O1)(C)C)/CO | -9.57941 |
| 255 | CS(=O)(=O)/C=C/C(=O)NCCC1=CC=C(C(=C1)O)OC/C=C(/CCC=C(C)C)\C | -10.3156 |
| 256 | CS(=O)(=O)/C=C/C(=O)NCCC1=CC=C(C(=C1)O)OC/C=C(/CC/C=C(\C)/CO)\C | -10.6677 |
| 257 | CS(=O)(=O)/C=C/C(=O)NCCC1=CC=C(C(=C1)O)OC/C=C(/CC/C=C(/C)\CO)\C | -10.6677 |
| 258 | CS(=O)(=O)/C=C/C(=O)NCCC1=CC=C(C(=C1)O)OC/C=C(/C[C@@H](C=C(C)C)O)\C | -10.5945 |
| 259 | CS(=O)(=O)/C=C/C(=O)NCCC1=CC=C(C(=C1)O)OCC[C@@H]([C@@H](CC=C(C)C)O)C | -10.9783 |
| 260 | CS(=O)(=O)/C=C/C(=O)NCCC1=CC=C(C(=C1)O)OC/C=C(/CC(=O)C=C(C)C)\C | -10.2708 |
| 261 | CS(=O)(=O)/C=C/C(=O)NC[C@@H](C1=CC=C(C=C1)OC/C=C(/CCC=C(C)C)\C)O | -10.3195 |
| 262 | CS(=O)(=O)/C=C/C(=O)NC[C@@H](C1=CC=C(C=C1)OC/C=C(/CC/C=C(\C)/CO)\C)O | -10.4727 |
| 263 | CS(=O)(=O)/C=C/C(=O)NC[C@@H](C1=CC=C(C=C1)OC/C=C(/CC/C=C(\C)/C=O)\C)O | -10.309 |
| 264 | O=S(=O)(C)/C=C/C(=O)N(CCC1=CC=C(C=C1)OC/C=C(/CC[C@@H](C(=C)C)OO)\C)C | -9.87278 |
| 265 | O=S(=O)(C)/C=C/C(=O)N(CCC1=CC=C(C=C1)OC/C=C(/CC[C@@H](C(=C)C)O)\C)C | -9.97418 |
| 266 | O=S(=O)(C)/C=C/C(=O)N(CCC1=CC=C(C=C1)OC/C=C(/C/C=C/C(C)(C)OCC)\C)C | -9.62161 |
| 267 | O=S(=O)(C)/C=C/C(=O)N(CCC1=CC=C(C=C1)OC/C=C(/C/C=C/C(C)(C)OO)\C)C | -9.66326 |
| 268 | O=S(=O)(C)/C=C/C(=O)N(CCC1=CC=C(C=C1)OC/C=C(/CC[C@@H](C(C)(C)OCC)O)\C)C | -10.7275 |
| 269 | O=S(=O)(C)/C=C/C(=O)N(CCC1=CC=C(C=C1)OC/C=C(/CC[C@@H](C(C)(C)O)OC(=O)C)\C)C | -10.6348 |
| 270 | O=S(=O)(C)/C=C/C(=O)N(CCC1=CC=C(C=C1)OC/C=C(/C[C@@H](C=C(C)C)O)\C)C | -10.0241 |
| 271 | O=S(=O)(C)/C=C/C(=O)N(CCC1=CC=C(C=C1)OC/C=C(/C[C@@H](C=C(C)C)OCC)\C)C | -9.79051 |
| 272 | O=S(=O)(C)/C=C/C(=O)N(CCC1=CC=C(C=C1)OCC[C@@](CC(=O)C=C(C)C)(C)O)C | -10.743 |
| 273 | O=S(=O)(C)/C=C/C(=O)N(CCC1=CC=C(C=C1)OCC[C@@](CC(=O)C=C(C)C)(C)OC)C | -10.1515 |
| 274 | O=S(=O)(C)/C=C/C(=O)N(CCC1=CC=C(C=C1)OC/C=C(/CC(=O)CC(C)C)\C)C | -9.94433 |
| 275 | O=S(=O)(C)/C=C/C(=O)N(CCC1=CC=C(C=C1)OC/C=C(/CC(=O)CC(C)(C)O)\C)C | -10.6105 |
| 276 | O=S(=O)(C)/C=C/C(=O)N(CCC1=CC=C(C=C1)OCC/C(=C/C(=O)C=C(C)C)/C)C | -9.63402 |
| 277 | O=S(=O)(C)/C=C/C(=O)N(CCC1=CC=C(C=C1)OC[C@@H](/C(=C/C(=O)C=C(C)C)/C)O)C | -9.97145 |
| 278 | O=S(=O)(C)/C=C/C(=O)N(CCC1=CC=C(C=C1)OCC/C(=C/C(=O)CC(C)(C)OC)/C)C | -10.21 |
| 279 | O=S(=O)(C)/C=C/C(=O)N(CCC1=CC=C(C=C1)OCC/C(=C\C(=O)C=C(C)C)/C)C | -9.66183 |
| 280 | O=S(=O)(C)/C=C/C(=O)N(CCC1=CC=C(C=C1)OCC/C(=C\C(=O)CC(C)(C)O)/C)C | -10.5213 |
| 281 | O=S(=O)(C)/C=C/C(=O)N(CCC1=CC=C(C=C1)OCC/C(=C\C(=O)CC(C)(C)OC)/C)C | -10.21 |
| 282 | C(=O)(/C=C/S(=O)(=O)C)NCCC1=CC=C(C=C1)O | -10.0536 |
| 283 | C12=C(C(=C3C(=C1OC)C=CC=C3)OC)C=CO2 | -9.39497 |
| 284 | C12=C(C(=O)C3=C(C1=O)C=CC=C3)C=C(O2)C(C)(C)O | -10.196 |
| 285 | [C@]12([C@H](C(=O)C3=C(C1=O)C=CC=C3)C[C@H](O2)C(O)(C)C)CC=C(C)C | -10.7756 |
| 286 | CC(=CC(=O)NCCC1=CC=C(C=C1)OC/C=C(/C1=CC(=O)C(O1)(C)C)\C)C | -9.5704 |
| 287 | CC(C)CC(=O)NCCC1=CC=C(C=C1)OC/C=C(/C1=CC(=O)C(O1)(C)C)\C | -10.0691 |
| 288 | C1=CC(=C(C=C1)NC)C(=O)NCCC1=CC=CC=C1 | -8.71516 |
| 289 | C1=CC(=C(C=C1)NC)C(=O)NCCC1=CC=C(C=C1)OC | -9.41073 |
| 290 | OC1=CC=C(C=C1)/C=C/C(=O)NCCC1=CC=C(C=C1)O | -9.00783 |
| 291 | CC(CC(=O)N(/C=C\C1=CC=CC=C1)C)C | -9.19263 |
| 292 | CC(=CC(=O)N(/C=C\C1=CC=C(C=C1)OC)C)C | -9.7398 |
| 293 | CC(=CC(=O)N(/C=C\C1=CC=CC=C1)C)C | -8.92221 |
| 294 | CC(=CC(=O)N(/C=C/C1=CC=CC=C1)C)C | -8.92221 |
| 295 | C1=CC(=CC=C1)C(=O)N[C@@H](C(=O)N[C@@H](COC(=O)C)CC1=CC=CC=C1)CC1=CC=CC=C1 | -9.53556 |
| 296 | C(=O)(C1=CC=CC=C1)O | -9.41121 |
| 297 | C(=O)(C1=CC=C(C=C1)O)O | -10.1885 |
| 298 | C(=O)(C1=CC=C(C(=C1)O)OC)O | -10.4679 |
| 299 | COC1=CC=C(C=C1)/C=C/C | -9.41897 |
| 300 | C1C(=O)C=C2[C@H](C1(C)C)CCC(=C2)CO | -10.4841 |
| 301 | C1[C@@H](C[C@@]2(C(=CC(=O)O2)C1(C)C)C)O | -10.4429 |
| 302 | CC(CC(=O)O)C | -9.13982 |
| 303 | C1(=CC=C(C=C1)/C=C/C(=O)OC[C@@]1([C@H]([C@@H](OC1)O[C@@H]1[C@H]([C@@H]([C@H](O[C@H]1OC1=CC(=C(C=C1)O)OC)CO)O)O)O)O)O | -10.7262 |
| 304 | C12=C(NC3=C1C=C(C(=C3)OC)C)C=CC1=C2C=CC(O1)(C)C | -10.9943 |
| 305 | C12=C(NC3=CC=C4C(=C13)C=CC(O4)(C)C)C(=C(C(=C2)C)O)[C@@H]1[C@H]([C@@](CO1)(C)O)C1=CNC2=C1C=CC=C2CC=C(C)C | -8.83099 |
| 306 | C(=C\C1=CC2=C(C(=C1)OC)O[C@H]([C@@H]2CO)C1=CC=C(C(=C1)OC)O)/C(=O)OC[C@@]1([C@H]([C@@H](OC1)O[C@@H]1[C@H]([C@@H]([C@H](O[C@H]1OC1=CC(=C(C=C1)O)OC)CO)O)O)O)O | -9.90258 |
| 307 | C1[C@@H](C[C@@H]([C@](C1(C)C)(/C=C/C(=O)O)O)C)O | -10.8079 |

Table S2. Redocking validation of co-crystallized ligands in ALK crystal structures and the corresponding RMSD values.

| **PDB ID** | **Co-crystallized ligand** | **Superimposition of crystallographic and redocked poses** | **RMSD (Å)** |
| --- | --- | --- | --- |
| 2XP2 | 3-[(1R)-1-(2,6-dichloro-3-fluorophenyl)ethoxy]-5-(1-piperidin-4-yl-1H-pyrazol-4-yl)pyridin-2-amine |  | 0.456 |
| 3AOX | 9-ethyl-6,6-dimethyl-8-[4-(morpholin-4-yl)piperidin-1-yl]-11-oxo-6,11-dihydro-5H-benzo[b]carbazole-3-carbonitrile |  | 0.822 |
| 4CCB | 3-[(1R)-1-[5-fluoranyl-2-(1,2,3-triazol-2-yl)phenyl]ethoxy]-5-(3-methyl-1H-pyrazol-4-yl)pyridin-2-amine |  | 0.587 |
| 4CCU | 2-(5-(6-amino-5-((R)-1-(5-fluoro-2-(2H-1,2,3-triazol-2-yl)phenyl)ethoxy)pyridin-3-yl)-4-methylthiazol-2-yl)propan-2-ol |  | 0.731 |
| 4CMO | 2-[(1R)-1-{[3-amino-6-(2-methoxypyridin-3-yl)pyrazin-2-yl]oxy}ethyl]-4-fluoro-N-methylbenzamide |  | 0.938 |
| 4CNH | **3**-[(1R)-1-(5-fluoro-2-methoxyphenyl)ethoxy]-5-(1-methyl-1H-1,2,3-triazol-5-yl)pyridin-2-amine |  | 1.395 |
| 4CTB | (5R)-8-amino-3-fluoro-5,19-dimethyl-20-oxo-5,18,19,20-tetrahydro-11,7-(azeno)pyrido[2',1':2,3]imidazo[4,5-h][2,5,11]benzoxadiazacyclotetradecine-14-carbonitrile |  | 0.346 |
| 4CTC | (10R)-7-amino-3-cyclopropyl-12-fluoro-1,10,16-trimethyl-16,17-dihydro-1H-8,4-(metheno)pyrazolo[4,3-h][2,5,11]benzoxadiazacyclotetradecin-15(10H)-one |  | 0.253 |
| 4MKC | 5-chloro-N~2~-[5-methyl-4-(piperidin-4-yl)-2-(propan-2-yloxy)phenyl]-N~4~-[2-(propan-2-ylsulfonyl)phenyl]pyrimidine-2,4-diamine |  | 1.695 |
| 5AAA | 3-[(1R)-1-(2,6-dichloro-3-fluorophenyl)ethoxy]-5-(1-piperidin-4-yl-1H-pyrazol-4-yl)pyridin-2-amine |  | 1.180 |
| 5AAB | 3-[(1R)-1-(2,6-dichloro-3-fluorophenyl)ethoxy]-5-(1-piperidin-4-yl-1H-pyrazol-4-yl)pyridin-2-amine |  | 1.377 |
| 5AAC | 3-[(1R)-1-(2,6-dichloro-3-fluorophenyl)ethoxy]-5-(1-piperidin-4-yl-1H-pyrazol-4-yl)pyridin-2-amine |  | 1.078 |

Table S3. Umbrella sampling and experimental binding free energies for co-crystallized ligands in PDB complexes 2XP2, 3AOX, 4CCB, 4CCU, 4CMO, 4CTC, 4MKC, 5AAA, 5AAB, 5AAC, 4CTB, and 4CNH

| **PDB ID** | **Co-crystallized ligand** | **ΔG_US_ (kcal/mol)** | **ΔG_exp_ (kcal/mol)** |
| --- | --- | --- | --- |
| 2XP2 | 3-[(1R)-1-(2,6-dichloro-3-fluorophenyl)ethoxy]-5-(1-piperidin-4-yl-1H-pyrazol-4-yl)pyridin-2-amine | -8.96 | -10.9196 |
| 3AOX | 9-ethyl-6,6-dimethyl-8-[4-(morpholin-4-yl)piperidin-1-yl]-11-oxo-6,11-dihydro-5H-benzo[b]carbazole-3-carbonitrile | -11.7 | -12.3695 |
| 4CCB | 3-[(1R)-1-[5-fluoranyl-2-(1,2,3-triazol-2-yl)phenyl]ethoxy]-5-(3-methyl-1H-pyrazol-4-yl)pyridin-2-amine | -10.4 | -10.2429 |
| 4CCU | 2-(5-(6-amino-5-((R)-1-(5-fluoro-2-(2H-1,2,3-triazol-2-yl)phenyl)ethoxy)pyridin-3-yl)-4-methylthiazol-2-yl)propan-2-ol | -8.11 | -10.7348 |
| 4CMO | 2-[(1R)-1-{[3-amino-6-(2-methoxypyridin-3-yl)pyrazin-2-yl]oxy}ethyl]-4-fluoro-N-methylbenzamide | -9.13 | -11.0571 |
| 4CTC | (10R)-7-amino-3-cyclopropyl-12-fluoro-1,10,16-trimethyl-16,17-dihydro-1H-8,4-(metheno)pyrazolo[4,3-h][2,5,11]benzoxadiazacyclotetradecin-15(10H)-one | -8.29 | -11.6821 |
| 4MKC | 5-chloro-N~2~-[5-methyl-4-(piperidin-4-yl)-2-(propan-2-yloxy)phenyl]-N~4~-[2-(propan-2-ylsulfonyl)phenyl]pyrimidine-2,4-diamine | -13.6 | -13.7563 |
| 5AAA | 3-[(1R)-1-(2,6-dichloro-3-fluorophenyl)ethoxy]-5-(1-piperidin-4-yl-1H-pyrazol-4-yl)pyridin-2-amine | -12.4 | -13.3293 |
| 5AAB | 3-[(1R)-1-(2,6-dichloro-3-fluorophenyl)ethoxy]-5-(1-piperidin-4-yl-1H-pyrazol-4-yl)pyridin-2-amine | -10.3 | -11.2191 |
| 5AAC | 3-[(1R)-1-(2,6-dichloro-3-fluorophenyl)ethoxy]-5-(1-piperidin-4-yl-1H-pyrazol-4-yl)pyridin-2-amine | -8.56 | -10.2061 |
| 4CTB | (5R)-8-amino-3-fluoro-5,19-dimethyl-20-oxo-5,18,19,20-tetrahydro-11,7-(azeno)pyrido[2',1':2,3]imidazo[4,5-h][2,5,11]benzoxadiazacyclotetradecine-14-carbonitrile | -7.88 | -10.4201 |
| 4CNH | **3**-[(1R)-1-(5-fluoro-2-methoxyphenyl)ethoxy]-5-(1-methyl-1H-1,2,3-triazol-5-yl)pyridin-2-amine | -8.95 | -9.95986 |

**Figure S1.** Schematic workflow of the integrated computational screening protocol, including compound collection and preparation, machine learning-based prediction, molecular docking, molecular dynamics simulation, steered molecular dynamics, umbrella sampling and potential of mean force calculation, and toxicity prediction.

|  |  |
| --- | --- |
| **2XP2** | **3AOX** |
|  |  |
| **4CTC** | **4CNH** |
|  |  |
| **4MKC** | **5AAA** |
|  |  |
| **5AAB** | **4CCU** |
|  |  |
| **4CTB** | **4CCB** |
|  |  |
| **4CMO** | **5AAC** |

Figure S2. Potential of mean force (PMF) profiles along the reaction coordinate (ξ) for co-crystallized ligands in complexes with PDB IDs 2XP2, 3AOX, 4CTC, 4CNH, 4MKC, 5AAA, 5AAB, 4CCU, 4CTB, 4CCB, 4CMO, and 5AAC, obtained from umbrella sampling simulations. The red curves represent the mean free-energy profiles, while the error bars estimated by bootstrapping method with 200 resampling rounds.

|  |  |  |
| --- | --- | --- |
| **t = 0 ps** | **t_max_ = 138.23 ps** | **t = 500 ps** |
|  | | |
| **53** | | |
|  |  |  |
| **t = 0 ps** | **t_max_ = 113.55 ps** | **t = 500 ps** |
|  | | |
| **54** | | |
|  |  |  |
| **t = 0 ps** | **t_max_ = 177.11 ps** | **t = 500 ps** |
|  | | |
| **95** | | |
|  |  |  |
| **t = 0 ps** | **t_max_ = 195.08 ps** | **t = 500 ps** |
|  | | |
| **160** | | |
|  |  |  |
| **t = 0 ps** | **t_max_ = 158.92 ps** | **t = 500 ps** |
|  | | |
| **164** | | |
|  |  |  |
| **t = 0 ps** | **t_max_ = 125.6 ps** | **t = 500 ps** |
|  | | |
| **165** | | |

Figure S3. Representative snapshots illustrating the dissociation pathway of studied compounds during the SMD simulation. Structures correspond to the initial configuration (t = 0 ps), the maximum-force event (t_max,_ ps), and the final dissociated state (t = 500 ps). The corresponding force–coordinate profile is shown.

|  |  |
| --- | --- |
| **53** | **54** |
|  |  |
| **95** | **160** |
|  |  |
| **164** | **165** |

Figure S4. Potential of mean force (PMF) profiles along the reaction coordinate (ξ) for compounds 53, 54, 95, 160, 164, and 165 obtained from umbrella sampling simulations. The red curves represent the mean free-energy profiles, while the error bars estimated by bootstrapping method with 200 resampling rounds.
